# Advancement in Human Face Prediction Using DNA

**DOI:** 10.3390/genes14010136

**Published:** 2023-01-03

**Authors:** Aamer Alshehhi, Aliya Almarzooqi, Khadija Alhammadi, Naoufel Werghi, Guan K. Tay, Habiba Alsafar

**Affiliations:** 1 Department of Biomedical Engineering, Khalifa University of Science and Technology, Abu Dhabi P.O. Box 127788, United Arab Emirates; 2 Center for Biotechnology, Khalifa University of Science and Technology, Abu Dhabi P.O. Box 127788, United Arab Emirates; 3 College of Medicine and Health Sciences, Khalifa University of Science and Technology, Abu Dhabi P.O. Box 127788, United Arab Emirates; 4 Department Electrical Engineering and Computer Science, Khalifa University of Science and Technology, Abu Dhabi P.O. Box 127788, United Arab Emirates; 5 Division of Psychiatry, Faculty of Health and Medical Sciences, The University of Western Australia, Crawley, WA 6009, Australia; 6 School of Medical and Health Sciences, Edith Cowan University, Joondalup, WA 6027, Australia; 7 Emirates Bio-Research Center, Ministry of Interior, Abu Dhabi P.O. Box 389, United Arab Emirates

**Keywords:** single nucleotide polymorphism (SNP), forensic DNA phenotyping (FDP), face landmarks, genome wide association studies (GWAS)

## Abstract

The rapid improvements in identifying the genetic factors contributing to facial morphology have enabled the early identification of craniofacial syndromes. Similarly, this technology can be vital in forensic cases involving human identification from biological traces or human remains, especially when reference samples are not available in the deoxyribose nucleic acid (DNA) database. This review summarizes the currently used methods for predicting human phenotypes such as age, ancestry, pigmentation, and facial features based on genetic variations. To identify the facial features affected by DNA, various two-dimensional (2D)- and three-dimensional (3D)-scanning techniques and analysis tools are reviewed. A comparison between the scanning technologies is also presented in this review. Face-landmarking techniques and face-phenotyping algorithms are discussed in chronological order. Then, the latest approaches in genetic to 3D face shape analysis are emphasized. A systematic review of the current markers that passed the threshold of a genome-wide association (GWAS) of single nucleotide polymorphism (SNP)-face traits from the GWAS Catalog is also provided using the preferred reporting items for systematic reviews and meta-analyses (PRISMA), approach. Finally, the current challenges in forensic DNA phenotyping are analyzed and discussed.

## 1. Introduction

During the last two decades, various genotyping techniques have been used to discover genetic factors responsible for variations in human appearance. Face character prediction has been a challenge for anthropologists, medical human geneticists and criminalists. In this review this type of prediction will be referred to as forensic DNA phenotyping (FDP). FDP aims to infer the unknown, externally visible characteristics (EVCs) of a person from DNA. After the anthropologists have established the bases of the phenotypes for human identification purposes, geneticists carry out research into genetic variations involving morphological features commonly used in human identification, such as age, ancestry, eye color, hair color, skin color, and facial features [1,2,3,4,5]. In 1996, Charles H. Brenner published a paper regarding the extension of the use of DNA short tandem repeat (STR) profiles to estimate the likelihood ratio of racially distinguishing Caucasians from African-Americans based on Bayesian reasoning [6]. Accordingly, the mathematical grounds for determining the ancestry of suspects who leave biological evidence at a crime scene was established. In addition, the determination of the inferential sense of human physical appearance such as ancestry and other phenotypes using DNA testing was discussed in a book by Tony Frudakis in 2008, using the term molecular photofitting [7]. DNA-phenotyping techniques can be significant in disaster victim identification (DVI), wherein facial identification of deceased individuals is difficult due to decomposition-induced changes in the skin, eye color, and other environmental factors. Such common disasters include tsunamis and hurricanes [8]. In addition, this type of facial prediction is vital in cases where searches in DNA and fingerprint databases and employing crime-scene clues have been exhausted without identification [9]. In 2003, genetic ancestry testing was first used to identify the race of a suspect behind a series of rape and murder cases in South Louisiana. Analyzing the evidence using a DNA Witness kit revealed that the suspect was of a mixed ancestry which in particular was (85%) African and (15%) Native American. This DNA profile led to the identification and conviction of the suspect in 2004 [10]. As the technology has advances and its reliability increased, it can now provide additional support to the traditional DNA-profiling methods. By providing an informative description of a suspect’s physical features, the technique can accelerate the investigation through the possible inclusion or exclusion of suspects based on the provided data [3,11,12]. In addition, when a victim’s skull is not available for facial reconstruction, victim identification can be hindered [13].

This review is organized as follows; in Section 1, we provide a general overview of DNA phenotyping. Section 2 exposes the different 2D and 3D facial scanning techniques and analysis tools. Section 3 provides an overview of the current face-landmarking techniques, algorithms, and analysis tools. In Section 4, a detailed survey of some approaches for analyzing and understanding facial features from DNA is provided. Section 5 elaborates on the present challenges in forensic DNA phenotyping.

## 2. DNA Phenotyping

The genetic influence on facial features has been investigated through studying various factors such as the impact of Sonic-Hedgehog, bone morphogenetic proteins, and homeobox genes on facial feature development [14,15,16,17]. Moreover, some genetic disorders, such as Neurofibromatosis, Fetal Alcohol Spectrum Disorder, the deletion of 22q11.2, and chromosomal abnormalities such as Down Syndrome can cause changes in facial features when compared to healthy individuals [18,19,20].

Epigenetic factors such as DNA methylation have shown reproducible results in age prediction due to the association between DNA methylation levels and age at some CpG sites [21,22,23,24]. DNA methylation levels in other genes, such as *ELOVL2*, *FHL2*, *KLF14*, *C1orf132/MIR29B2C*, and *TRIM59*, were also correlated with a mean absolute deviation (MAD) of 3.844 years from chronological age [25]. On the other hand, Xia et al. used three-dimensional facial image analysis to predict the age of Chinese participants with an average difference between the predicted and chronological age of only ±2.8 to 2.9 years [26]. This finding demonstrates the importance of age in face prediction, along with other genetic factors.

In addition, examining the European population within datasets such as the 1000 Genomes Project showed that polygenicity strongly affects phenotypes. Nevertheless, there is a correlation between different phenotypes within specific groups of people as they share similar genetic variations [27,28]. There is a link between facial traits and population substructures, which suggests that facial morphology could be affected by ancestry [17]. Most of the studies included in the genome wide association studies (GWAS) Catalog on genetic-to-phenotype associations were conducted on populations of European descent with an underrepresentation of other populations, such as the Middle Eastern population, which only contributed to 0.08% of the GWAS database [29,30]. Studying samples from under-represented population groups can improve our knowledge of genetic structures and extend the applicability of forensic and medical findings [31].

Ancestry-based SNPs (AISNP) are usually linked with facial traits because the predictability of a given population’s ethnicity is higher when the population’s facial features are more distinct [32].

Research reports have demonstrated the importance of AISNPs in forensic, medical, and anthropological applications [33,34,35]. The Kidd and Seldin AISNP panels include diverse data concerning reference populations from major continental regions [33]. Most of the ancestry markers are di-allelic (insertion–deletion) makers or SNPs, as the current method of using short tandem repeat (STR) markers does not predict ancestry. Hence, SNPs can provide investigative leads [36]. The Snipper App Suite is an open-source tool that provides multiple solutions for biogeographical classifications based on massive parallel sequencing (MPS) panels that contain AISNPs [37,38]. Other commercial kits have also been developed to determine ethnic background and ancestry, including AncestryDNA, 23andMe, and National Geographic [39,40,41].

AISNPs have different frequencies in different populations, thus enabling the determination of individuals’ ancestry using DNA [33,42,43]. An individual’s genetic ancestry is mainly presented as a proportional ancestry or admixture by determining the population most correlated with the genetic variation in the DNA sample [44,45,46].

In addition to the investigative leads that AISNPs can provide, phenotype-informative SNPs (PISNPs) reveal more information regarding a suspect’s physical appearance. Such phenotypes include the color of the subject’s eyes, hair, and skin, as well as their facial features [5,47,48,49,50,51,52,53,54]. There are complex interactions between the genes controlling the phenotypes of individuals, such as mutations, genetic drift, recombination, segregating variants, and copy number variants. Therefore, scientific collaborators from genetics, image-processing engineering, bioinformatics, statistics, and other backgrounds have focused on categorizing the genetic variations correlated with a specific phenotype during the last decade. This collaboration aims to understand the aforementioned correlation and accurately predict facial features [17,55].

One of the main categories that identifies and distinguishes an individual from another is color. The prominent, primarily identifiable visible colors are related to a person’s eyes, hair, and skin. One of the tools designed to predict these three phenotypes is the HIrisPlex-S system, which combines prediction models for eye, hair, and skin color [51,56].

The IrisPlex eye color prediction tool demonstrated a prediction accuracy of over 90% with respect to blue/brown phenotypes using only 31pg of DNA when applied to Dutch Europeans, making this kit sensitive and suitable for applications in low-copy-number DNA samples [57,58,59,60]. However, the tool shows low prediction accuracies for green-hazel or intermediate dark phenotypes and admixed populations, thus indicating the need to further investigate the tool using admixed populations and larger sample sizes [61,62,63]. Another GWAS study incorporating many European participants (~193,000) from 10 population groups identified 124 genetic loci for eye prediction, of which 50 had not been reported previously. The findings also demonstrated consistencies in the gene structure of the eye color traits between East Asian populations and Europeans [64]. In addition, some researchers have tested the tool on the Pakistani population using degraded DNA samples, which showed lower accuracies (70%), mainly due to the considerable variation of phenotypic eye color in Europeans compared to South Asians. They recommended refraining from using the tool if some SNPs had dropout alleles [65].

Regarding hair color prediction, the HIrisPlex tool showed an area under the curve (AUC) between 72–92 for blond, brown, red, and black hair colors, which indicates the potential of the tool for applications in the forensics [5,47,48,49,50,52].

SNPs for skin color prediction were included in the HIrisPlex-S tool and were distributed among 16 pigmentation genes. Two skin tone models were assessed using the following two approaches: three and five skin tone scales. The use of the three skin tone models (light, dark, and dark-black) demonstrated an accuracy ranging from 83–97%, while five skin tone models ranging from very pale to dark-black showed an accuracy range of 72% to 97% [51,53]. Some of the genes in the HIrisPlex-S tool were further studied in admixed populations and indicated similar associations [66,67,68,69]. In addition, the association of the SNP rs12913832 (*HERC2*) with the three skin pigmentation traits was discovered in Polish, European, and mixed populations, including Hispanics [70,71,72,73]. Furthermore, a GWAS study conducted on 17,019 Korean women revealed associations of seven loci with face pigmentation, of which three had not been previously reported [74]. Other researchers are testing these multiplexes and try to discover their associations to the nearby genes. Their purpose is to validate and optimize the accuracy of their results when applied to different populations [75,76,77].

On the other hand, Arab researchers have tested some of the primary loci associated with eye color, such as *HERC2* and *OCA2*, on a Middle Eastern population (Iraqi). They discovered that due to a linkage disequilibrium between the SNPs of these loci and the variations of the minor allele frequencies, some deviations from the model with respect to predicting dark-brown, hazel, and blue eye colors were discovered since the tools did not account for Middle Eastern populations [78]. Other researchers also confirmed similar outcomes in other Middle Eastern populations (Saudi and Iranian) [72,79]. This shows the importance of validating prediction tools on multiple population groups in order to understand the resulting variations before officially using the tool in forensic applications.

Overall, eye, hair, and skin color prediction showed different accuracy levels among population groups and lower prediction accuracies for intermediate color groups, which can be improved by increasing the genetic markers that account for ancestry and by expanding studies on admixed populations [5,47,49,50,51,53,56,72,80,81,82]. The HIrisPlex-S tool is an open-source tool that is available online for any forensic investigators who are interested in obtaining an inference of hair/skin/eye color using the allele copy number of the specified SNPs of interest. The software provides the accuracy level of the results based on AUC values [83].

### Inference of Face Features

The use of the human skull to reconstruct an entire face has been employed in the forensic investigation of the deceased bodies for the last few decades. Facial photos could be highly important for identifying unknown individuals. These images were successfully used in identifying unknown individuals [84,85,86]. In 2004, Turner et al. developed reality enhancement/facial approximation by computational estimation (RE/FACE) software to predict a skull’s soft tissue structure. This software was created for the Federal Bureau of Investigation (FBI) to automatically employ dense landmark placement using computerized tomography (CT) scans. Recently, other open-source computerized tools such as FacIT were developed to allow for the reconstruction of a person’s face by scanning their skull using tools such as CT [13,87]. These tools have helped investigators and anthropologists identify people from different eras and population groups, despite the controversy. Some researchers wanted to explore the influence of DNA on the skull, and their findings suggest that facial traits are composed of variations in cranium and soft tissue thickness [88].

To address instances where there is no skull from which to build a face, facial trait inference is currently being researched using DNA-phenotyping techniques. This method provides investigative leads regarding a person’s physical appearance from biological evidence. Similar to face reconstruction from skulls, facial landmarks are essential in this type of analysis. They can be used to distinguish faces through linear measurements between them, such as the face height, nose (width, prominence, and size), interocular distance, chin and forehead prominence [5,47,48,49,50,51,89,90].

One of the approaches that investigated the effect of gender, ancestry, and genetic variations on facial measurements used bootstrapped response-based imputation modeling (BRIM) to measure and model facial shape variations. The related study involved 592 participants from an admixed population of West Africans and Europeans. The sample pool was obtained from the United States, Brazil, and Cape Verde. They found 24 SNPs distributed among 20 genes that significantly affect face morphology. Moreover, from the total number of 7150 high-density quasi-landmark (QL) configurations of the superimposed and symmetrized 3D faces, 44 principal components (PCs) were selected, which described 98% of the total variation [80].

Other GWAS studies demonstrated the strong association between genetic variations and facial features such as face width, forehead protrusion, cheek protrusion, nose ridge elevation, nasal length and protrusion, nasion position, nose bridge breadth, and the distance between the eyeballs. These associations were mostly presented as *p*-values and standard deviation values rather than as a percentage of accuracy. Each study targeted a different population, which may have affected the correlation values due to factors affecting ancestrally correlated facial features. Optimizing such technology for forensic applications requires a further understanding of the genetic bases of human appearance. Such optimization relies on the use of larger sample sizes from different population groups, understanding the related epigenetic factors, investigating possible environmental factors, involving collaborators of varying scientific disciplines, and improving analysis methods [1,8,17,89,91,92,93,94,95].

## 3. Facial Screening and Scanning Tools

### 3.1. Facial Screening Using 2D Approach

Two-dimensional images of the face have been used in clinical applications for the last decade (ferry et al., 2014). Different medical applications have incorporated 2D photographs in the diagnosis of genetic syndromes and face-related anomalies at earlier stages of the human development [96]. The establishment of highly sophisticated centers with advanced equipment and technologies is very difficult in some rural areas and poor communities. The use of phones offers a readily available method and can provide the information required by geneticists to offer their diagnoses online. This type of inference will create a practical option for the early diagnosis of children from underdeveloped areas. Moreover, many datasets that are available online, such as Face2Gene and Ferry and Colleagues, have been made public to allow researchers to incorporate them into their tools [97,98]. As a result, the algorithms has been simplified to be trained on face photos of patients of various genetic conditions [99].

The architecture of the deep learning algorithm was created by developing three levels of neural networks, which first standardize the 2D face images, then detect the shape of the face, and, finally, estimate the genetic syndrome risks. Based on 2800 children’s photos from different countries, age and gender were used to transform the ability for phone photos and phone-based applications to be used as primary genetic screening tools. In general, the model evaluated the risk of children presenting with a genetic syndrome with an average accuracy of 88%. This study shows the amount of information a face can reveal about an individual’s genetic makeup using 2D photographs. The system required the manual landmarking of the faces at 44 locations. Then, measurements between these landmarks based on an in-house application were taken as each quantity of dysmorphology was associated with a specific genetic syndrome. Since photographs cannot provide details in (mm) units, the interpupillary distance for every patient was used as a standard to normalize error.

These technologies have higher success rates when the genetic diseases/syndromes involve joint deformation morphologies of the face, such as in Williams–Beuren, Crnelia de Lange, Down’s, 22q11.2 deletion, and Noonan syndromes [96].

However, to acquire a higher level of facial detail for human identification purposes and to better represent the depth of a face, the third dimension is required. Most researchers recommend using 3D scanners. They add higher resolution and more accuracy in capturing facial details [100]. Thus, the following section focuses on the use of 3D face scanning techniques.

### 3.2. 3D Face-Phenotyping Techniques

Three-dimensional surface imaging refers to the technique wherein three-dimensional data are acquired from an object as a function of three coordinates (*x*, *y*, and *z*). Three-dimensional scanners generate exact point clouds by obtaining an object’s fine details and capturing free-form shapes. Thus, once these features are transformed into digital data, they can be used for different purposes, such as quality checks and measurements. Moreover, surface imaging mainly works through measurements of coordinate points on the surface of an image. These measurements can be viewed as a depth map function (*z*) of the position (*x*, *y*) in the Cartesian coordinates system. They can also be expressed in a matrix form {*zij* = (*xi*, *yj*), *i* = 1, 2, …, *L*, *j* = 1, 2, …, *M*} [101,102].

Different technologies are used in 3D scanners, in which each technique serves an assorted purpose and has its advantages and disadvantages. These technologies include phone applications, laser triangulation, structured light, and stereophotogrammetry.

#### 3.2.1. Advanced Phone Application in 3D Scanning

The practice of gathering a sequence of points in space from a series of images is known as photogrammetry. First, multiple 2D images of the object from all possible angles are required; then, the software will connect all the relevant points from the overlapping process of these images and create a 3D mesh [103]. The latest innovations by mobile technology companies such as Apple, Sony, and Samsung have made it possible to generate 3D photos using their devices. iPhone models such as 12 Pro, 13 Pro, or the newest iPad Pro may use LiDAR scanning. These devices are equipped with built-in LiDAR sensors, which enable them to easily scan oversized objects using depth data. Multiple types of software and applications utilize such technologies to process photos and create 3D objects, such as Trnio and Scann3d. In addition, such phones can use augmented reality (AR) to 3D-register physical objects with exceptional accuracy. While these techniques are promising, these phones will need to have between 20–40 different photos of an object to acquire an acceptable scan [104]. The Trnio 3D scanning software has two configurations: object and scene modes. After the photographer scans the object, the software provides immediate assistance in these modes. For the object mode, the photographer moves around the object and the application creates a panoramic photo of the object to be available in a circular manner. Scene mode is for free scanning, which means it can be used to scan massive objects or outdoor scenes in 3D.

Other options may require the use of plug-in devices along with the phone, such as itSeez3D and Bevel [105]. These devices are usually beneficial for lower phone capabilities as they provide extra sets of cameras and eye-safe lasers as detectors. The collected information is analyzed with software that collects size and geometric information from the laser, while it collects the color, texture, and other object features from the phone camera [104].

Although smart-phone applications are promising and have successfully recreated 3D-printed household objects, the high number of photos required and the long time it takes to create a scan can lower the accuracy especially in forensic related research.

#### 3.2.2. Laser Triangulation-Based 3D Scanners

These scanners scan an object either by a laser line or a single laser point. The scanner emits the laser, and its light gets reflected off the scanned object. First, a sensor targets the initial trajectory. Based on the changes between the trajectory and the angle of triangulation, the system perceives specific aberration angles. These angles are associated with the distance between the scanner and the object. When sufficient distance measurements are collected, the scanner maps that object’s surface, thus creating a 3D picture. The scanner can also be used on moving objects, as it can collect a series of profiles from the laser lines that form a complete 3D map of an object. However, triangulation-based scanners exhibit safety issues concerning the safety of the participants’ eyes [106]. They may also perform poorly with respect to the scanning of shiny objects and materials with significant subsurface scattering [107,108]. This method was utilized in capturing 3D face scans of the participants of the Avon Longitudinal Study of Parents and Children (ALPAC) [109,110]. This laser constituted eye-safe technology accepted by the U.S Food and Drug Administration (FDA) with a wavelength of 690 nm at 30 mW. Using this type of scanner shows how important it could be to revisit older technologies and attempt to improve them for possible applications in facial scanning.

#### 3.2.3. Structured Light 3D Scanners

This type of scanner uses active illumination. It flashes 2D spatially assorted varying-intensity patterns generated by a special light source or a projector. Then, it obtains the object’s surface information using an imaging sensor. Suppose the light is projected onto a planar surface (i.e., 2D surface). In that case, the pattern acquired by the camera will be similar to the projected pattern, which means there will be no distortion of the projected structured light. However, if the surface is nonplanar, which means it has prominence and depth, the projected structured light will be distorted. The distortion pattern can be computed using different algorithms to generate the 3D surface of the object. These scanners can be either handheld or stationary. Usually, these scanners are made from static SLR cameras and a light projector such as the SL2 system produced by XYZ RGB Inc. (Kanata, ON, Canada) [101,111]. Revopoint 3D Technologies Inc. (Xi’an and Shenzhen, China) produces other methods that use the same technique, including the Handysense handheld 3D scanner [112].

#### 3.2.4. Stereophotogrammetry 3D Scanners

The concept of these scanners involves the production of 3D images from a series of 2D images using computer vision algorithms. In this method, several photographs are taken of the object from different viewpoints using any accessible camera. The changes from one photo to the next are calculated via algorithms that automatically recognize pixels corresponding to the same physical point, resulting in a 3D image. This technology can scan objects of various scales [113]. Canfield Scientific Inc. (Parsippany-Troy Hills, NJ, USA) is a company that invented the VECTRA^®^ M3 system based on the stereophotogrammetry 3D scanner model. Plastic surgeons mainly use this system to view high-resolution images of the face and neck [114].

#### 3.2.5. Selecting the Right Type of Scanners

When choosing a 3D scanner for capturing facial features, several factors need to be considered. The foremost factors are the scanning resolution and the accuracy. Scanning resolution is the smallest distance between two points on the object that the scanner can measure. Accuracy is the degree to which the measured value conforms to the object’s actual value. Moreover, as the distance between the scanner and the object increases, the absolute value of the error increases accordingly, and this is a value that should be considered. When dealing with human subjects and reflective objects, laser triangulation systems may harm the subjects’ eyes if suitable wavelengths are not considered. To compare the three types of systems, four devices were selected in terms of their popularity of use in 3D-based facial research (Table 1).

Konica Minolta Vivid 900 laser cameras have the highest 3D resolution, the smallest file size, and the fastest processing speed among the four devices. The 3dMDhead system, a stereophotogrammetry stationary device, that can scan in 360 degrees the entire face, head, and neck. This device allows the user to capture frames at the highest speed. The 3dMDhead system was used in multiple forensically related papers, as shown in Table 1. The other device that uses stereophotogrammetry is Vectra H1 from Canfield. It has the lowest geometry resolution, at 0.8 mm, and the lowest accuracy, at 0.84, among the other instruments. The vertices of the images created by this device are the highest, with a 1.2 mm resolution.

Moreover, Vectra H1 can be purchased at the lowest price compared to the other two devices (about half of their respective prices). On the other hand, the Artec Eva system is a handheld device that requires rotation around the object while capturing the pictures; thus, a great deal of time is required to capture a face (~4 min processing time per person). It also requires more effort from the subject to stabilize their facial expression and the person using the device to maintain a specific distance range from the subject to achieve an adequate scan. Overall, the four scanners are suitable for capturing facial details. Selecting the proper device depends on the nature of its application and the target price, resolution, coverage, and accuracy. From the literature, the 3dMDhead scanner seems to be more favorable among face-related studies due to its high speed with respect to capturing details and the consistency of the results between the collected data and the facial measurements of the subjects. However, this scanner is costly and requires a high level of experience to set up. Moreover, it requires a designated room to work at a high level of accuracy.

In the coming future, the use of phones to take 2D/3D photos could advance face–DNA research in the forensic, anthropological, and medical fields. If accuracy and precision were benchmarked against the discussed scanners, researchers would have practical options with which to conduct their investigations, generating fewer expenses and smaller setup areas. This option will positively impact the study of facial traits in the forensic field as it has for the emerging genetic medical diagnosis tools.

## 4. Face Landmarks, Algorithms, and Analysis Tools

### 4.1. Face Landmarks

A face landmark can be defined as a prominent discriminative position on the face that can be used as a reference point for facial comparison. The selection of landmarks depends on the phenotype being investigated, but most of these landmarks lie in the oral-nasal region of the face. Face landmarks were introduced in 1994 by the pioneer of modern craniofacial research Leslie Farkas, who suggested modeling faces using 17 landmarks [128].

Face landmarks are grouped into either primary or secondary groups. The primary (first-order) landmarks consist of the nose tip, corners of the mouth, corners of the eyes, etc. They focus on forensic applications, including the major features used in human identification. The localization of the primary landmarks can be carried out using tools such as the histogram of gradient (HOG) and scale-invariant feature transform (SIFT) [129]. In addition, the secondary (second-order) landmarks are guided by the primary landmarks because they represent points that have more details regarding non-extremity points. These landmarks usually represent features such as nose saddles, chin tips, and cheek curves, which are typically used to understand facial expressions or when analyzing one-sided/incomplete facial photos.

Some issues can be encountered when obtaining a high-quality facial scan, which involve poses, expression, illumination, occlusions, etc. [129]. Landmarking approaches include manual, semi-automated, fully automated, and face masking using quasi-landmarks. An overview of these methods is provided in this review.

### 4.2. Manual Landmarking

Measurements between the landmarks are obtained by taking anthropometric linear measurements. Weinberg et al. compared direct anthropometry using calipers, 2D photogrammetry from photos, and cephalometry of the skull using radiography techniques. It was shown that these techniques could not accurately capture the details of 3D human faces [128,130].

The placing of landmarks is usually performed by marking the face with points using a marker and measuring distances between them or estimating landmark positions without using a marker. This process can be performed manually using digital or ruled calipers. These measurements can also be obtained by uploading the facial scans on software that allows for 2D photo and 3D face mesh rotations [128]. It is preferable to use software to obtain such measurements due to the uncontrollable nature of the manual identification of landmarks and the possibility of a higher degree of error through operators’ intra- and inter-variations [127,128,131,132,133]. Multiple statistical analyses have been performed in order to investigate the precision of each technique (manual caliper vs. 3D image using a reference of dots versus without dots). The mean absolute difference, relative error magnitude, practical error of measurement, and the coefficient of consistency of each landmarking technique were compared. The results showed higher accuracy when the 3D images were marked using computer software [128].

### 4.3. Semi-Automated Landmarking

The semi-automatic landmarking of facial features can be performed using MATLAB^®^ (Natick, MA, USA) and in-house developed software, including image analysis tools. Viola-Jones is one of the tools that use object-detection algorithms to detect faces and eyes, while an Active Appearance Model annotates the remaining landmarks. An operator supervises this process and confirms the annotated landmarks created by the automated system [93]. Another approach to semi-automatic landmarking is to generate 500 points of uniformly spread digitized landmarks using a sliding technique. The points are generated from a template 3D mesh that initially contains 16 manually selected landmarks. The points then radiate from the template landmarks to ensure a uniform spread on the facial surface (approximately 1.5 mm radius). This sliding of landmarks is expected to increase the accuracy of geometric analysis as it includes extra characteristics of the face, such as the curves and Procrustes distance. This technique is performed using multiple programs such as Viewbox (Kifissia, Greece) [134] and RStudio (Boston, MA, USA) packages (the geomorph package) [135,136].

### 4.4. Automated Landmarking

Since manual landmarking is time-consuming and challenging to replicate, automating the process can help deal with large databases and cover larger face areas. Therefore, different statistical models and algorithms have been developed to detect facial features and automatically place landmarks. Some techniques use the geometric properties of a face’s surface by incorporating one or multiple of the following differential geometry techniques: mean, Gaussian, principal curvatures, different shape indices, and curvedness [137]. One method involved the use of a thresholding technique to examine the correlation between the location of each landmark and the behavior of each predefined geometric descriptor on the face [138]. These methods were effective in localizing/detecting segments of the face without expressions or occlusion.

Moreover, landmarks were successfully extracted from 3D faces with standard, expressive, and occluded mouth/eyes using MATLAB^®^ algorithms [138,139]. The success rate of the facial feature extraction algorithms was tested on available face databases such as FRAV3D, FRGC 1.0 and FRGC 2.0, Face Warehouse, and GavabDB [140,141,142,143]. This study demonstrated the possibility of building a reliable, automated landmarking method even with faces with different expressions and occlusions. There are also multiple available online datasets of 3D faces/scans used to evaluate the accuracy and efficiency of new methods, as the performance of algorithms has been shown to decrease when a test is performed with another dataset [129].

Another technique for automatic landmarking used a statistical ensemble approach based on comparing magnitudes of complex Gabor wavelet coefficients to the dataset. The algorithm improved the accuracy of landmarking facial scans by up to 22%. It was found that the stacking generalization algorithm of facial features can decrease the average error to 1.7 mm across 21 landmarks. The model was also successful in training the algorithm using the minimal number of 3D facial images, and it was able to handle large cohort GWAS studies [144,145]. It is important to note that most of these methods are under development and have not been intensively used in genetic studies [146]. In one of the GWAS studies, 2D facial photographs were utilized by obtaining metric measurements after converting the pixels from the photos of the faces into millimeters via different algorithms [146].

### 4.5. Estimation of 3D Face Landmarks Using Mobile Devices

Multiple solutions are available for the detection and estimation of 3D face landmarks using 2D photos taken by phones operating on Android and IOS platforms. One of these programs is named MediaPipe Face Mesh [147]. In real time, this application can estimate 468 LMs of the face. It can screen the position of the face through face transformation within a space by bridging the gaps between the estimated LMs. The machine learning pipeline is built based on a neural network model that detects the full-face image in order to align and connect the frames and another one that approximates a 3D face using regression models [147].

### 4.6. Face Masking and Quasi-Landmarks

Some researchers have established a guide to map the 3D face meshes in question to a template that resembles an anthropometric mask [148]. The open-source MeshMonk tool is a script designed to automatically quantify the dense surfaces of the biological phenotypes in question. It automates the orientation and resizing of the face, maps the face using a non-rigid transformation to a spatially dense face anthropometry model, and then uses the so-obtained modality in multivariate statistical analysis [149]. This mapping also establishes correspondences between the quasi-landmarks from the model and mesh points from the targeted faces. To account for changes in the orientation, positioning, and size scale of the face, Generalized Procrustes Analysis (GPA) was used. This superimposition was performed to combine the original and reflected configurations of the faces. This creates an asymmetric and bilaterally symmetric component for each decomposed shape of the face. The difference in asymmetry was determined from the average symmetrical component of the reflected configuration. By using these steps, differences between facial asymmetries were ignored, and the only components used were the symmetrical ones [150]. Multiple researchers have confirmed that the accuracy of using 3D facial scans with automated measurements was better than direct anthropometry [127,128]. Researchers have tested the accuracy of the MeshMonk tool on 41 human faces and found it to be similar to the use of 19 manual landmarking placements with an average Euclidean distance error of 1.26 mm and a range of 0.7–1.68 mm [151].

Some computer programs such as Cliniface include tools for the automatic extraction of facial landmarks from 3D facial scans [100,149]. This software uses the MeshMonk algorithms to landmark 69 points of a 3D face. In addition, linear and angular facial measurements can also be obtained using the same software [100,149]. This tool is of great use since it is available online for free and is designed for dysmorphological facial analysis research. Furthermore, the software provides a high level of accuracy with respect to extracting the measurements from 3D faces by comparing the results to manual extraction performed by an expert [100].

Most researchers in forensic and anthropological fields use linear distance measurements. These measurements can allow for greater collaboration between different study groups, especially when 3D face datasets cannot be shared due to ethical research restrictions set to protect the volunteers [91].

## 5. Current advances in Approaching Genetically Based 3D Facial Shape Analysis

The nature of EVC inference using DNA is complex due to environmental factors that can affect facial feature formation. Thus, larger 3D facial datasets are required to begin exploiting the capabilities of this technology. Furthermore, FDP techniques and analytical approaches are in their development stages, and greater agreement is required among the scientific community concerning the best approaches [149,152]. Sero et al. presented a framework for research on modeling facial features based on average faces, and then retouched using DNA [124]. The framework consists of three stages: unraveling genetic architectures, perceptual analysis and applications, and the predictive modeling of faces. The model also lists the disciplines needed for each stage, mainly comprising biologists, geneticists, bioinformaticians, image analysts, forensic scientists, lawyers, and policymakers. There are currently two approaches to studying facial features using genetics: the DNA-to-face approach and the face-to-DNA approach [124].

### 5.1. DNA to Face Approach

The DNA-to-face approach is a mode of investigation used by geneticists to understand the phenotypic aspects of the face based on genetic mutations using DNA-genotyping techniques [153]. The method is also investigated using enhancer activity detection by a reporter gene assay and RNA-seq assays in non-human models such as mice and zebrafish [154,155].

**Figure 1:**
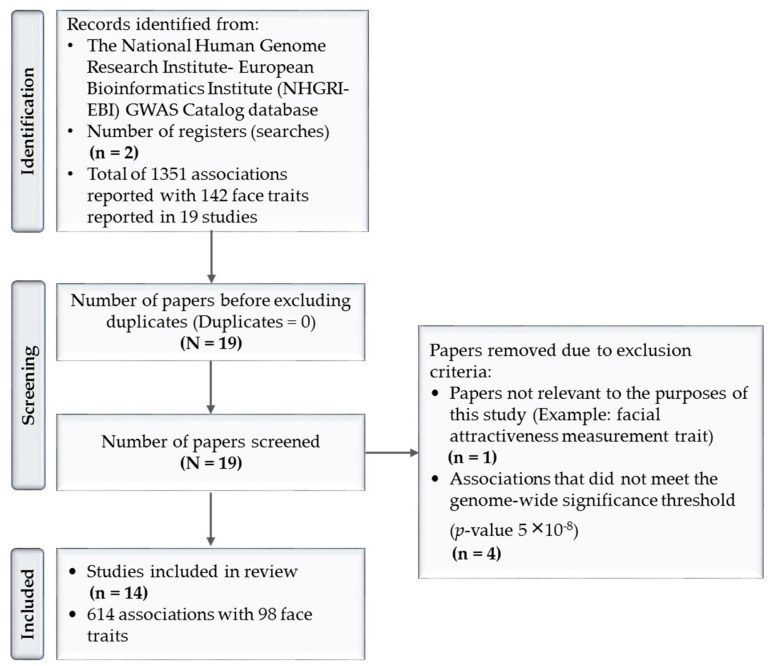
Literature review stages using PRISMA approach.

The GWAS investigated the association between SNPs and facial features, age, and genomic ancestry. Forensic scientists have been focused on DNA-phenotyping research, especially in the last ten years. Due to the abundantly generated data, multiple databases have been established to organize the related research findings. For the purposes of this study, The National Human Genome Research Institute–European Bioinformatics Institute (NHGRI–EBI)’s GWAS Catalog database was used to report the SNPs that have been significantly associated with facial features to date. This database is one of the main resources of GWAS studies and its tools provided the bases to identify, exclude, and include the studies of this review, as detailed in (Figure 1) [156].

The results shown in the identification stage of (Figure 1) were obtained by first searching the register “facial morphology measurement” trait in the GWAS Catalog on 30 April 2022. Facial morphology measurement was described as the “quantification of some aspect of facial morphology” such as “lip thickness”, “forehead height”, or “chin protrusion” [156]. The results included 1101 SNP associations with 109 traits in 19 publications (*p*-value 1 × 10^−79^–9 × 10^−2^) [91,146,154,157,158,159,160,161,162,163,164,165,166,167,168,169,170,171,172]. However, this search did not reveal facial features regarding the nose area. As a result, another search was conducted on the “nose morphology measurement” trait, which was described as the “quantification of some aspect of nose morphology”, such as “nose wing breadth”, “nose tip shape”, or “nose profile” [156]. A total of 250 associations resulted from this search, with 33 nose traits in 11 studies [146,154,157,158,160,161,162,163,165,168,169]. From these two searches, a total of 1351 associations with 142 facial traits were reported in a total of 19 GWAS studies [91,146,154,157,158,159,160,161,162,163,164,165,166,167,168,169,170,171,172]. A total of five papers were excluded [91,157,166,167,172].

**Figure 2:**
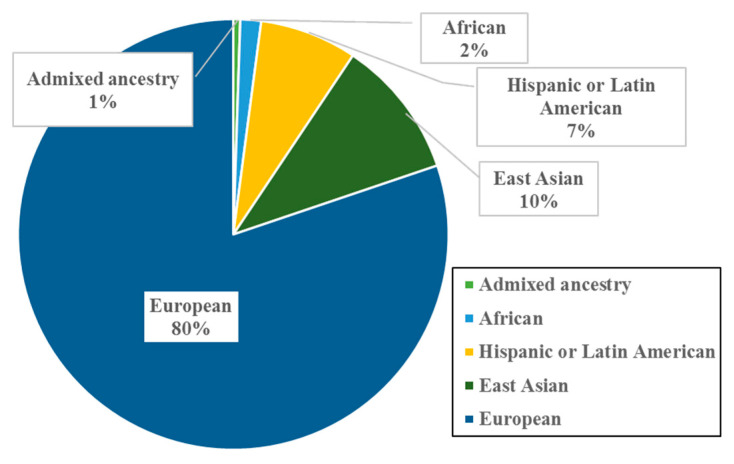
Population ancestries in 19 research studies investigating SNP-face morphology [91,146,154,157,158,159,160,161,162,163,164,165,166,167,168,169,170,171,172]. These percentages include both discovery and replication samples.

Analysis of population backgrounds in these papers revealed that most of the studied populations were of European ancestry (80%), followed by East Asians (10%), Hispanic or Latin Americans (7%), Africans (2%), and those of Admixed ancestry (1%) (Figure 2). Such results support the persistent European bias in GWAS data, which was previously reported in 2016 [30].

**Figure 3:**
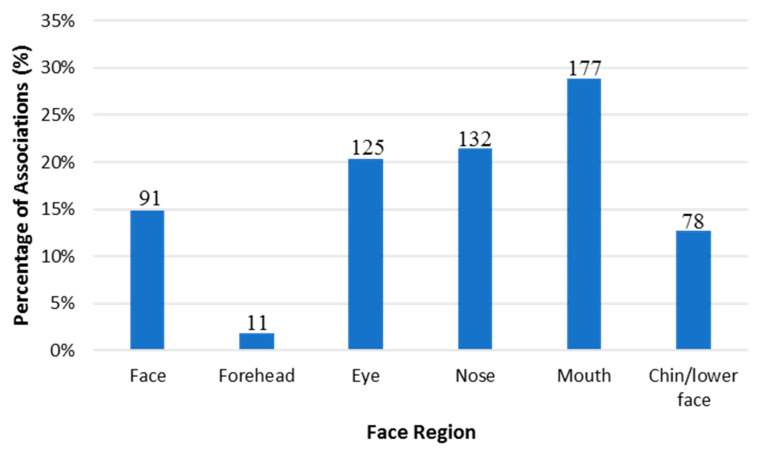
Percentage of SNPs that met the GWAS significance threshold in the GWAS Catalog according to six facial regions (Face, forehead, nose, mouth, lip, and chin/lower face). “Face” indicates traits that were associated with multiple facial regions. Numbers on each bar reflect the number of the associations for each facial region.

For the purposes of this review, the “facial attractiveness measurement” trait was excluded due to its non-relevance to facial feature prediction using DNA. In addition, only SNPs that met the GWAS *p*-value threshold of (5 × 10^−8^) or higher were reported. This threshold was selected based on the Bonferroni correction method. This method accounts for the *p*-value based on the assumption that every SNP is tested independently of an array [173]. It considers the linkage disequilibrium (LD) that may present between SNPs of the same array. As a result, the calculation considers that there are, based on the genome, 1,000,000 possible LD. Thus, 0.05/1,000,000 will yield this threshold (*p* < 5 × 10^−8^) [174]. This is considered one of the most conservative methods for selecting the *p*-value threshold [174,175]. In addition, note that the database reports one *p*-value for the correlated trait, which may be the *p*-value of discovery, replication, or meta-analysis. Therefore, the database was used as a filtering tool for all SNPs that reached the genome-wide significance threshold. A total of 614 associations with 98 traits (*p*-value 1 × 10^−79^–5 × 10^−8^) met our inclusion criteria (Tables S1–S6). Figure 3 demonstrates the distribution of these associations according to six facial regions (facial traits affecting multiple areas of the face, forehead, nose, mouth, lip, and chin/lower face). Most of the associations were found in the mouth area (29%), followed by the nose (21%), eye (20%), face (15%), chin/lower face (13%), and forehead (2%).

When assessing genes of two or more associations with facial features, inconsistent patterns were observed within and between the population groups (Table 2). Some genes were associated with features in the same facial region in different publications on the same population group. For instance, the PABPC1L2A and PABPC1L2B genes were associated with intercanthal width in two studies conducted on the European population [154,165]. PABPC1L2 encodes for binding protein cytoplasmic, and among its related pathways are mRNA surveillance pathways and RNA transport [176]. Similarly, two studies conducted on individuals of European ancestry reported associations of the NAV3 gene with mouth morphology measurements [165,169]. The NAV3 gene is a member of the neuron navigator family and is mainly expressed in the nervous system [177].

In contrast, some genes showed associations with different facial features within the same population group. For example, the transmembrane 74 (*TMEM74*) gene was associated with the height of the vermillion lower lip in the European population [165]. This gene was also associated with features in the nose segment in another study conducted on the same population group [169]. In addition, several studies suggest the involvement of the *TMEM74* gene in tumor cell survival through the induction of autophagy in multiple tumor cell lines [178,179].

When investigating the associations between different population groups, some genes showed consistent associations with features in the same facial region, while others showed inconsistent findings. For instance, the *PAX3* gene was associated with nasion position in the Hispanic/Latin American individuals [158] and with features in the nose segment in the European population [169]. The *PAX3* gene plays a critical role during fetal development and is involved in normal bone development in the skull and face [180]. Similarly, the regulator of chondrogenesis RNA (ROCR) gene was associated with five nose traits in different population groups, such as profile nasal angle, nasal tip protrusion, and nasolabial angle in East Asians [146], and nose size and traits in the nose segment in two studies conducted on Europeans, respectively [162,169]. The *ROCR* gene has a biased expressed mainly in the salivary gland [181].

Other findings suggest the association of genes in different face areas among different population groups. The *SUPT3H* gene was associated with forehead protrusion in Hispanic/Latin Americans [161], nose morphology measurements in East Asians [160], and chin dimples and the nose segment in Europeans [162,169]. The *SUPT3H* gene is related to pathways involved in transcriptional misregulation in cancer and chromatin-folding patterns [182]. It is also associated with several diseases, including Cleidocranial Dysplasia, which is a rare genetic disorder that affects tooth and bone development [183]. Another gene, *HDAC9*, was associated with an indication of columella in Hispanic/Latin Americans [161], while it was associated with mouth morphology measurement in Europeans [165]. *HDAC9* (Histone deacetyltransferase 9) is an enzyme engaged in regulating gene expression. Although *HDAC9* is not expressed in the craniofacial tissues of developing mice, it is proposed that it regulates the expression of *TWIST1*, a neighboring gene affecting limb and craniofacial development in mice [161].

The above observations suggest the involvement of multiple genes in face morphology. In addition, some of these genes affect traits in the same facial region within and between population groups, while others have inconsistent patterns. These findings indicate the complex interaction in gene–face morphology, and it is essential to conduct additional studies to understand such associations and how other anatomical and developmental factors affect variations between different ancestries.

### 5.2. Face to DNA Approach

Naturally, a person can distinguish between feminine and muscular facial features. In addition to the symmetry characteristics between male and female faces, the differences are statistically significant [184]. Moreover, face-shape differences between phylogenetically related populations were shown to be statistically significant [185]. The phenotype-to-genotype approach, also known as the face-to-DNA approach, uses the average face. It is generated based on each gender and genomic ancestry. The face is then remodeled/modified based on multiple SNPs that have previously been associated with specific facial features. The modifications on the faces are performed using machine learning tools, 3D facial scan databases, genetic traits, and other human biometric authentication measures [168].

Another investigated aspect in this approach constitutes face-to-DNA classifiers, which is a labeling approach that categorizes given faces into different classes based on molecular features. The algorithm used by the authors of [124] generates 7150 QLs based on wrapping a templated average face over the given face. Afterward, a squared similarity matrix is constructed using the RV coefficient between each pair of QLs configurations. First, the segments are arranged hierarchically using hierarchical spectral clustering. Then, these segments are divided multiple times until the total number of facial areas reaches 63 face segments. This dense surface registration tool targets specific 3D facial features to aid the performance of statistical analysis, classification, regression, score fusion, and biometric evaluations, as well as the discovery of new associations between phenotypes and genotypes. Using samples grouped into two primary study cohorts (Global and European), full, 3D faces were segregated into 63 segments/modules based on the ethnicity of the cohort. The preselected SNPs were generated from the GWAS 9.5 million SNPs, in which 1932 SNPs were positioned at 38 separate markers. Using this approach, the authors demonstrated 83% and 80% verified matches in the global and European cohorts [124].

Other researchers have also studied the approach of phenotype-based genomics. They were able to compare different models to test the effects of including some SNPs and genetic factors related to age, ethnicity, gender, height, body mass index (BMI), vocal pitches, and other parameters in the prediction algorithms used for identification. The prediction accuracy with respect to facial structure was enhanced when BMI and age were considered. Facial features were predicted using multiple types of algorithms, such as PC, linear discriminant, neural networks, sparse representation, and the local presentation of facial features. Their approach was mainly to deform the face and map it against a template and calculate the displacements between them to overcome the challenge regarding data privacy in personalized medicine. Using 1000 ancestry PC data, the algorithm predicts the face’s PC value based on ridge regression and multiple covariates such as sex, age, and BMI. This algorithm was developed based on maximum entropy in order to combine phenotypic features and GWAS. Future studies shall include populations of different ethnic groups in order to validate the current research outcomes and explore facial traits that are less common among individuals of European ancestry [186].

Considering the differences between the two approaches, the authors of [187] tested the possible improvement in predicting hair structure, freckles, and the color of hair, skin, and eyes using trait-prevalence-informed priors. The model of the priors was based on including biogeographical ancestry groups in a Bayesian framework. They compared this model to the previously proposed DNA-based (prior-free) EVC-predicting models. The priors model showed minimal effect on the prediction of some facial features, while it did not affect others. This study suggests that using prevalence priors, similar to the face-to-DNA approach, may not be the right approach to understanding the EVC. The researchers recommend focusing on the genetic factors directly affecting the facial traits independently from the population’s genetic factors [187]. However, considering the difference between the well-established pigmentation prediction method and the complex genetic architecture of facial feature prediction, additional traits ought to be assessed by the trait-prevalence-informed priors model. These features include nose protrusion, nose length, eye curvature, chin depression, etc.

### 5.3. Statistical Approaches

Considering the complexity of the process of facial prediction, applying the power of machine learning techniques, algorithms, and other statistical models to the big data available can alter the approaches to the challenge at hand. Although simple, linear models are considered great classifiers when aiming to avoid overfitting. There are regularized linear models, such as maximum likelihood, and those with less regularization using variable selection simultaneously, such as lasso, which is a preferred model for increasing accuracy in high-dimensional data [188,189]. Using the right training data based on a known variable, the supervised learning model can be targeted for each type of trait. In general, quantitative traits that involve measurements are usually approached using regression. On the other hand, when analyzing categorical traits that involve pigmentation (more than two categories), the multi-class classification supervised learning model is considered [190]. As an example, the polygenic score model—employing weighted allele sums of multiple SNPs—was used to predict height-related features [189]. The area under the receiver operating characteristic curve (AUC) and R^2^ were used to calculate the general performance or the accuracy of prediction models [189].

In addition, researchers have used partial-least-squares regression (PLSR) for predicting face-related features based on genomic ancestry, sex, and 24 SNPs [80]. Others used ridge regression of 1000 genomic principal components along with ancestry and sex genomic factors, which were coupled with age and BMI, to increase accuracy [187]. In addition, a shape-similarity statistic that used the shape space angle between 3D faces was used in PCA and PLSA models with 277 SNPs [168]. There are also other models that are considered black-box models which utilize ensemble, decision trees, and neural networks methods. Most of these networks are deep and have hidden layers of combined signals that are trained mostly by gradient or back-propagation algorithms [190]. Choosing the right features regarding the learning rate of neural networks, their layers, and the neurons available within them can help optimize such algorithms for prediction. Since the face is considered a complex, non-linear, continuous model, continuous latent features are better approached using variational autoencoders. In these methods, building a cost-effective prediction model is dependent on selecting the effective features of the target variable. Some of the methods that are used to filter redundancies and connect variables—even in a non-linear manner—are the information theory models [190].

## 6. Challenges in Forensic DNA Phenotyping

Multiple challenges need to be addressed before applying FDP in forensic cases. First, the accuracy of FDP needs to be assessed, mainly when the result of this evidence is used for exclusion or conviction. Second, FDP raises ethical concerns by revealing medical information that the suspects/victims may not wish to know/have released. Third, FDP has been legislated in only a few countries, and the legal terms need to be updated in others. Fourth, some individuals make cosmetic or surgical alterations to their facial features, making FDP more challenging. Lastly, the generated FDP data and their analytical procedures need to be further assessed by forensic and research laboratories worldwide to obtain a valid scientific basis before using FDP as legal evidence in forensic cases.

### 6.1. Accuracy

The success of FDP can be measured by validating the accuracy of predicted facial features in real forensic cases. However, the current research on FDP focuses on associating genes with face-related phenotypes, which predicts a class of phenotypes but does not yet individualize one face from another [191]. Moreover, several aspects need to be addressed before using the technology in forensic cases, including its accuracy and the standardization of validation testing [192]. The accuracy of FDP can be advanced by identifying more gene-related phenotypes and conducting studies on larger population groups from different ethnicities [186,193].

### 6.2. Ethical Issues

As the research on FDP progresses, more information can be predicted using DNA. Initially, DNA was used to generate profiles for identification in forensic cases. These STR profiles were thought to be uninformative and were mainly used for identification purposes. However, research has shown that these genetic markers have a regulatory role in gene expression. For instance, Bañuelos, M., et al. demonstrated correlations between genotypes in the Combined DNA Index System (CODIS) loci and expression variations of the neighboring gene and, possibly, medical information [194]. Therefore, genetic privacy has become one of the major challenges for FDP because of the continuous improvements and advances in this technology [193]. Another aspect of privacy is the availability of the FDP data to third parties, thereby granting them power due to holding such information while making the individual vulnerable from a “knowledge is power” perspective [186,195].

Not all characteristics revealed by FDP have the same level of sensitivity. For instance, some characteristics are trivial and are not private, such as the external features of a subject, which includes voice type or right-handedness. In addition, police and law enforcement agencies have access to portrait photographs on drivers’ licenses and ID cards. On the other hand, medical history is characterized as a sensitive trait. If this trait is revealed to the public, it can be used as a filter criterion in the employment process. Moreover, it is argued that the advantages of the limited use of these features in criminal investigations do not override the privacy risks faced by the individual [195]. It is significant to note that although the same gene can code for both pathological and physical variations, the mutation causing the variation is different. For instance, the OCA2 gene codes for oculocutaneous albinism type 2. It also codes for variations in eye, skin, and hair color. However, the SNPs associated with each variation are different [1].

FDP can potentially reveal information regarding genetic diseases that individuals may not wish to be informed about, thus violating the “right not to know” principle. Regarding the human genome, United Nations Educational, Scientific and Cultural Organization (UNESCO), in Article 5c, declared that “The right of every individual to decide whether or not to be informed of the results of the genetic examination and the resulting consequences should be respected” [196]. Similar statements were declared in the Rights of the Patient approved by the World Medical Association, Patients’ rights in French law, the European Convention, the Human Genetics Advisory Commission (HGAC) in the United Kingdom, the Dutch Medical Treatment Act of 1994, the Hungarian Health Act of 1997, and the Belgian Patient’s Rights Act of 2002 [196]. Generally, it is believed that the advantages of identifying criminals and preventing them from committing more crimes exceed the benefits of preventing patient discrimination. This argument has affected the legal legislation of FDP in Texas state, which legalized the use of FDP, including in testing for diseases. On the other hand, other countries, such as the Netherlands, disallowed the use of disease-related information in forensic investigations in 2003 [1].

From another perspective, some argue that the weight of the right to maintain ignorance is dependent on the value of information revealed by FDP. For example, some traits do not reveal medical information and do not necessarily violate privacy rights, such as left-handedness, the tendency to smoke, voice type, or geographic origin. These traits are mostly previously known by the individual and will not raise an issue if confirmed by FDP [5,47,48,49,50,51,52]. In addition to the possibility of violating the “right not to know principle,” it is important to identify the circumstances of the collection and storage of FDP data, e.g., the decision of whether FDP analysis will be implemented in all forensic cases or whether it will be restricted to cases where a DNA match has not been found [197].

To address the data protection and privacy issues raised by individuals, it is suggested that FDP data are destroyed following the criminal investigation or when a match is found. This option ensures that the collected data are used for their original purpose, which is identification, and potentially eliminates the use of such information for other purposes. However, destroying FDP information may violate the Universal Declaration on the Human Genome, which gives the individual the right to know/be unaware of the result of a genetic test [196,198]. Further FDP-related ethical concerns include the storage of such data during and after investigations and access to FDP information, which need to be addressed by the scientific community before using this information as evidence in forensic investigations [199].

### 6.3. Bias

There is evidence of bias in the research into FDP. This bias mainly stems from the researchers or the machines used. Researchers may use easier-to-access populations to conduct their research, and they may try to obtain specific grants based on the population of interest. Accordingly, some populations, such as Europeans, are more prone to be included in human genetics research. In addition, since most researchers are from universities and research centers in well-developed countries, it will be easier to target populations around them rather than collect samples from other areas, which invokes logistical bias.

Moreover, it is easier for researchers to compare and use pre-established databases such as the GWAS Catalog. However, this will create persistent bias throughout the years because machine learning and the verification of the results are easier when using more data from the same subjects’ ancestry. Hence, there are many variables to control when introducing a new population and more to correct for before learning from novel populations. In addition, some machines that have already been developed may find it harder to compare and provide accurate results since the ancestry information available (genotypes) and phenotypic information—such as lifestyle, facial characteristics, and diet—vary between populations.

Hence, some technologies may not accurately present complete perspectives of phenotypic information if this bias is not considered. For example, light, shades of darker colors, and reflection are not well-considered by scanning machines that have already been developed using lighter skin-color populations. It is necessary to optimize these machines to gather the full potential of the data in order to reach higher levels of accuracy.

Some researchers have shown that GWAS is a great tool for discovering the genetic factors involved in complex diseases. Hundreds of thousands of significantly associated biological characteristics and genetic loci have been found. These associations have been of high value as they helped understand the biological mechanisms of diseases and other phenotypes. However, admixed populations or those of non-European ancestry are under-represented in the GWAS Catalog. Hispanic and Latin American ancestry, Pacific Islanders, Native Peoples, and Arab and Middle Eastern subjects comprised less than 1% of the catalog in the year of 2016. Arab and Middle Eastern populations have contributed to only about 0.08% of the whole GWAS dataset as of 2016. This continuation of bias will create many implications for research, such as (1) impairing the accuracy of findings if direct associations are used across populations, (2) hindering the discovery of novel genetic associations, and (3) limiting the understanding of the face-related genomics in the forensic, anthropological, and medical research on unexplored populations [30].

### 6.4. Legal Issues

FDP was recently introduced in forensic investigations. Therefore, some countries are still updating their laws regarding the use of FDP in forensic cases. Netherlands, Slovakia, and Germany are the only countries that legalized FDP for forensic purposes, while Belgium, Greece, France, Luxembourg, and Ireland prohibited the use of FDP [54,200]. While some argue that FDP acts as an eyewitness and thus does not require any legislation, others have restrictive views on the application of FDP in forensic investigations. For instance, some countries, such as South Africa, restricted FDP to non-coding markers, as most SNPs associated with facial features are located in the intronic region [1,195]. As an example, the rs2045145 SNP is intronic and is associated with the female European second PC extreme profile [121]. In addition, an intron of the *PARK2* gene (SNP rs9456748) was found to be significantly associated with the height of the midface [158]. Additionally, the intronic variant of *COL23A1* (collagen type 23 α 1) (SNP rs118078182) is associated with variations in the nasal shape of individuals across Eurasia [95].

As FDP technology advances, another approach to legislating the use of FDP in forensic investigations is to specify the forensic purpose, i.e., identification or 3D facial prediction, rather than restricting the use of certain markers [1]. Overall, most current DNA regulations are related to traditional DNA profiling, which is based on comparisons of DNA profiles obtained from reference and evidence samples. Consequently, FDP technology requires new legal considerations that are different from those applied to traditional DNA typing [201].

### 6.5. Facial Cosmetic Changes

The number of plastic surgeries and cosmetic procedures has exponentially increased worldwide. This causes a limitation that needs to be addressed, especially for technologies that are based on face recognition algorithms [202]. Cosmetic changes can be minor, such as fillers, skin lifts and OnabotulinumtoxinA injections, as well as plastic surgeries of the eyelids and those involving the reshaping of the nose. The effect of these procedures can be minor such as changes in skin texture or major such as changes in the natural measurements of facial features [203]. An evaluation of the current facial recognition algorithms showed lower performance when applied to faces that underwent plastic surgery [204]. This might be even more challenging for FDP studies as the genetic components cannot be accurately associated with the natural landmark positions or face measurements. Other temporary modifications include the application of hair dye, wearing colored contact lenses, and the use of tanning products. Such modifications are also expected from fugitives who try to avoid being recognized by law enforcement agencies.

Consequently, caution must be taken in FDP-guided investigations to avoid falsified appearances [1,205]. The common changes that people make to their natural appearance indicate that new FDP technologies need to be robust and accurate, even towards cosmetic changes. A new aspect of FDP research is the study of the extent of such modifications and their effects on the accuracy of newly developed techniques [206]. Overall, overcoming the limitations of facial 3D prediction using DNA can be achieved by increasing the volume of the EVC-related studies [5,47,48,49,50,51,52].

### 6.6. Evaluation and Validation

The evaluated research studies demonstrated that the prediction of ancestry and pigmentation traits such as skin, eye, and hair color is more developed than the prediction of facial features, indicating the need for additional research before accepting the use of this technology in forensic fields [62,193,200]. Extensive research is needed to precisely identify the genes associated with variations of each trait. The collaboration of research institutions, governments, private organizations, commercial companies, and the forensic laboratories of law enforcement agencies around the world is needed to conduct massive genome-wide association studies. In addition, intensive research is essential in order to establish a database of candidate genes associated with facial features. It is of high value to understand that a comprehensive database can greatly enhance the current state-of-the-art technologies used in forensic laboratories by supporting the transition of DNA profiling databases to a new technology that can potentially generate evidence without the need for reference samples. The use of these databases can be efficient by including many subjects, a wide age range, different facial expressions, and various ethnic groups [1,206]. Some available 3D face databases include FaceBase, Stirling ESRC 3D Face Database, Bosphorus Database, etc. [207,208,209,210]. In 2017, scientists developed the VISible Attributes Through GEnomics (VISAGE) Consortium to validate and enhance the application of FDP in forensic cases. This consortium brings together eight working groups to cover multiple disciplines related to FDP, such as the confirmation of genetic markers and statistical tools, cooperation with face-sketchers, the training of individuals of interest, the setting of policies, and the acquirement of required ethical approval. This collaboration aims to predict individuals’ ancestry, facial features, and age using the massive parallel sequencing of a large number of data [211,212].

Face base also serves as a hub for collaborators/researchers interested in craniofacial research. FaceBase has criteria for accepting and sharing facial data from different models (animals and humans) [213]. In addition, the European DNA Profiling Group (EDNAP) aims to assess the reliability and consistency of the currently available technologies used in forensic science by establishing meetings and comparing the data of laboratories around the world. Moreover, the reproducibility of the IrisPlex System has been validated by EDNAP across 21 laboratories, which has revealed its potential for success. However, since most genetic associations with face morphology are determined from homogeneous populations, additional studies are needed to validate such associations in admixed populations [52].

Although the promising results of FDP indicate that it could replace the current STR-profiling techniques, this replacement may not be feasible in the near future due to the large amount of money spent on the existing STR profiles and infrastructure in place in developed national databases worldwide. As a result, future genetic markers used for FDP will likely be added to the core STR markers without replacing the existing STR technology [92].

## 7. Discussion and Conclusions

The current state of the art regarding DNA-phenotyping techniques has been highlighted in this paper. Multiple technologies (scanning tools, software, algorithms, etc.) are available for acquiring facial feature measurements, which vary with respect to their ease of use and data accuracy. In the meantime, new technologies are evolving fast, reflecting the importance of revisiting the literature to remain informed of the latest technologies and algorithms developed in such research areas.

Most scientists believe that it is too early for DNA technologies to be fully employed in face prediction. Nevertheless, genetic studies have cleared the picture of the influence of genetics on facial morphology. With the use of GWAS on limited population groups, researchers have been able to identify some associations between genetic markers and facial features. However, facial analysis based on DNA differs from Mendelian diseases due to the multiple and complex factors affecting facial morphology. Thus, other approaches or combinations of them need to be considered.

Some challenges FDP encountered by include those related to assessing its accuracy before implementing it in forensic cases. In addition, applying practices to maintain FDP data privacy is fundamental, especially if such data reveals medical information. The regulation of access to FDP data and storage methods are essential in order to avoid information exploitation by third parties/less scientifically sophisticated police officers. Limited efforts have been made to address these concerns, which is understandable considering that it is an emerging technology, and its extent and validity are not fully established [211]. Moreover, the fitness of face inference algorithms or statistical tests can be improved by increasing the number of investigated individuals, conducting studies on underrepresented population groups, and identifying more face-related genetic markers. Lately, the discovery of homogeneous populations has been a challenge, as the migration of individuals has become easier, and intermarriage is more common across the world. For example, the percentage of intermarriage of newlyweds in the USA was 3% in 1967. The same percentage in 2015 has increased 4.7 times to reach 17% [213]. These admixed populations could affect the outcomes of prediction models and could increase the error rate if not corrected for via population stratification. Moreover, it is recommended that researchers investigate faces globally and locally by correlating grouped features or measurements with individual or multiple genetic factors [167]. Other factors to be included in FDP analysis include epigenetics, telomere lengths, and non-genetic factors [214,215].

## Figures and Tables

**Table 1:** A comparison between four scanning tools using three different 3D scanning techniques: 3dMDhead, Canfield VECTRA H1, Artec Eva, and Vivid 900 [115,116,117,118].

Model/Products	3dMDhead	Canfield VECTRA H1	Artec Eva	Konica Minolta Vivid 900 Laser Cameras (Mid Lens)
Realization	Active/Passive stereo photogrammetry	Passive Stereo Photogrammetry	Structured Light	Laser scan
Coverage	Full 360-degree capture of the head, face, and neck	Capturing volume (H × W × D): 220 × 130 × 70 mm typical application: 100-degree of left, right, or front of face.	Closest (H × W): 90 × 70 mm Furthest (H × W): 180 × 140 mm	Closest (H × V): 204.7 × 153.6 mm Furthest (H × V) 830.6 × 622.9 mm
3D Resolution	0.2 mm	0.8 mm geometric resolution (triangle edge length)	0.5 mm	0.016 mm
3D Point Accuracy	0.58 ± 0.11 mm	Average: 0.84 mm (range 0.19–1.54 mm)	0.1 mm 0.03% over 100 cm	X: ±0.38 mm, Y: ±0.31 mm Z: ±0.20 to the Z reference plane
Capture Speed	~0.0015 s at highest resolution	0.008 s	0.067 s/frame	0.3 s (fast mode)/2.5 s (Fine mode)/0.5 s (Color mode)
Processing Speed	<15 s	~20 s	4 min (for facial scans)	1 s (Fast) 1.5 s (Fine)
File Size	15–95 MB. Depends on configuration.	8 MB	10–20 MB (full-body scan ranges from 2–4 GB according to Artec3D technical support email)	1.6 MB (fast), 3.6 MB (Fine)
Geometric Representation	A continuous point cloud available as a textured mesh and densely textured point model	Mesh	Mesh	Original format converted to 3D by the utility software (640 × 480)
Error in Geometry	<0.2 mm	<0.1 mm	<0.1 mm	N/A
Approximate Price	Prices start at USD 25,700 (each system is costume-configured and upgraded from standard modules to meet the customer’s specific imaging workflow requirements)	USD 11,000	~USD 21,000	USD 25,000 to 55,000
Utilized by	[119,120,121,122,123,124]	[123,124,125,126]	[125,126]	[109,127]

**Table 2:** Genes that demonstrated two or more associations with facial traits from the Tables S1–S6 [146,154,158,160,161,162,163,165,169,170,171].

Number of Associations for Each Gene	Genes	Facial Region	Phenotypes	Ancestry	Reference
2	*TRPC6*	Face	Upper facial depth	European	[154]
			Middle facial depth	European	[154]
2	*LINC01470*	Face	Facial width measurement	European	[165]
	*LINC01470, PRKAA1*		Facial width measurement	European	[165]
2	*ZRANB2-AS2*	Face	Factor 13, vertical position of alar curvature relative to upper lip	European	[165]
			Factor 13, vertical position of alar curvature relative to upper lip	European	[165]
2	*TRPM1, LINC02352*	Face	Facial width measurement	European	[165]
			Middle facial depth	European	[154]
2	*RERE*	Eye	Right eyelid peak position ratio	East Asian	[146]
			Tangent line angle of er3	East Asian	[146]
2	*ATP8A1*	Eye	Upper eyelid sagging severity	European	[170]
			Upper eyelid sagging severity	European	[170]
2	*PABPC1L2A, PABPC1L2B*	Eye	Factor 14, intercanthal width	European	[165]
			Intercanthal width	European	[154]
2	*ZNF385D*	Eye	Upper eyelid sagging severity	European	[170]
			Upper eyelid sagging severity	European	[170]
2	*CACNA2D3*	Nose	Segment 52	African	[163]
			Nose size	European	[162]
2	*GLI3*	Nose	Segment 22	European	[169]
			Nose wing breadth	Hispanic/Latin American	[158]
2	*LINC00399, LINC00676*	Nose	Nose protrusion	Hispanic/Latin American	[161]
			Nose size	Hispanic/Latin American	[161]
2	*LINC00676*	Nose	Nose size	European	[162]
	*LINC00676, LINC00399*		Segment 20	European	[169]
2	*LINC01121, SIX2*	Nose	Columella size	Hispanic/Latin American	[161]
			Segment 44	European	[169]
2	*LINC01432*	Nose	Nostril size	Hispanic/Latin American	[161]
			Segment 54	African	[163]
2	*PAX3*	Nose	Nasion position	Hispanic/Latin American	[158]
	*PAX3, RPL23AP28*		Segment 11	European	[169]
2	*PAX7*	Nose	Columella inclination	Hispanic/Latin American	[161]
			Segment 11	European	[169]
2	*PKHD1*	Nose	Segment 11	European	[169]
	*PKHD1, FTH1P5*		Segment 22	European	[169]
2	*PRDM16*	Nose	Nose roundness 1	Hispanic/Latin American	[161]
			Nose size	Hispanic/Latin American	[161]
2	*RUNX2, SUPT3H*	Nose	Nose bridge breadth	Hispanic/Latin American	[158]
			Nose morphology measurement	East Asian	[160]
2	*LINC00620*	Mouth	Mouth morphology measurement	European	[165]
			Lower lip height	European	[154]
2	*LINC02820, RASSF9*	Mouth	Segment 30	African	[163]
			Segment 9	European	[169]
2	*NAPB*	Mouth	Factor 15, philtrum width	European	[165]
			Factor 15, philtrum width	European	[165]
2	*PCCA*	Mouth	Factor 5, width of mouth relative to central midface	European	[165]
			Factor 5, width of mouth relative to central midface	European	[165]
2	*NAV3*	Mouth	Mouth morphology measurement	European	[165]
			Segment 35	European	[169]
2	*NHP2P2, HOXA1*	Mouth	Segment 9	European	[169]
			Philtrum width	European	[171]
2	*SACM1L*	Mouth	Factor 5, width of mouth relative to central midface	European	[165]
			Labial fissure width	European	[154]
2	*SDK1*	Mouth	Factor 5, width of mouth relative to central midface	European	[165]
			Factor 5, width of mouth relative to central midface	European	[165]
2	*STXBP5-AS1*	Mouth	Lip protrusion	Hispanic/Latin American	[161]
			Lower lip protrusion	Hispanic/Latin American	[161]
2	*LINC01117*	Chin/Lower face	Chin dimples	European	[162]
			Segment 24	European	[169]
2	*LINC01965*	Chin/Lower face	Chin dimples	European	[162]
	*LINC01965, AHCYP3*		Jaw slope 2	Hispanic/Latin American	[161]
2	*CPED1*	Chin/Lower face	Jaw protrusion 2	Hispanic/Latin American	[161]
	*CPED1*		Jaw protrusion 5	Hispanic/Latin American	[161]
2	*RNU7-147P, PLCL1*	Chin/Lower face	Chin dimples	European	[162]
			Segment 53	European	[169]
2	*TNFSF12, TNFSF12-TNFSF13*	Chin/Lower face	Segment 26	European	[169]
			Chin dimples	European	[162]
2	*SEM1*	Chin/Lower face	Chin dimples	European	[162]
			Segment 54	European	[169]
2	*ADAM15*	Forehead	Segment 41	African	[163]
		Chin/Lower face	Chin dimples	European	[162]
2	*ADGRL4*	Face	Factor 9, facial height related to vertical position of nasion	European	[165]
		Mouth	Factor 5, width of mouth relative to central midface	European	[165]
2	*CLYBL*	Eye	Segment 59	African	[163]
		Mouth	Factor 5, width of mouth relative to central midface	European	[165]
2	*DENND1B*	Face	Factor 4, facial height related to vertical position of gnathion	European	[165]
		Chin/Lower face	Chin morphology	East Asian	[160]
2	*HDAC9*	Nose	Columella inclination	Hispanic/Latin American	[161]
		Mouth	Mouth morphology measurement	European	[165]
2	*KCNQ1*	Face	Factor 13, vertical position of alar curvature relative to upper lip	European	[165]
	*KCNQ1, KCNQ1OT1*	Mouth	Segment 9	European	[169]
2	*LINC01376*	Nose	Segment 22	European	[169]
	*LINC01376*	Chin/Lower face	Segment 24	European	[169]
2	MN1	Face	Middle facial depth	European	[154]
		Eye	Factor 8, orbital inclination due to vertical and horizontal position of exocanthion	European	[165]
2	*PRRX1, GORAB*	Chin/Lower face	Segment 51	European	[169]
	*PRRX1, MROH9*	Mouth	Segment 9	European	[169]
2	*RAD51B*	Nose	Nose size	European	[162]
	*RAD51B*	Mouth	Segment 17	European	[169]
2	*RN7SL720P, BNC2*	Nose	Columella size	Hispanic/Latin American	[161]
		Chin/Lower face	Chin dimples	European	[162]
2	*RPS27AP14, DMRT2*	Face	Factor 9, facial height related to vertical position of nasion	European	[165]
		Nose	Nose size	European	[162]
2	*TBX3, UBA52P7*	Eye	Segment 14	African	[163]
		Nose	Segment 5	European	[169]
2	*TMEM74*	Mouth	Factor 6, height of vermillion Lower lip	European	[165]
	*TMEM74, EMC2*	Nose	Segment 10	European	[169]
3	*VPS13B*	Nose	Columella size	Hispanic/Latin American	[161]
				East Asian	[160]
			Nasolabial angle	East Asian	[146]
3	*LSP1*	Mouth	Lip thickness 1	Hispanic/Latin American	[161]
			Lower lip thickness 1	Hispanic/Latin American	[161]
			Lower lip thickness 2	Hispanic/Latin American	[161]
3	*WARS2*	Mouth	Lower lip thickness 2	Hispanic/Latin American	[161]
			Lip thickness ratio 1	Hispanic/Latin American	[161]
			Lip thickness ratio 2	Hispanic/Latin American	[161]
3	*BMP7*	Nose	Segment 23	European	[169]
		Nose	Nose size	European	[162]
		Mouth	Factor 5, width of mouth relative to central midface	European	[165]
3	*C17orf67*	Face	Lower facial depth	European	[154]
	*C17orf67*	Eye	Factor 8, orbital inclination due to vertical and horizontal position of exocanthion	European	[165]
	*C17orf67, NOG*	Mouth	Segment 38	European	[169]
3	*CRYGFP, MEAF6P1*	Mouth	Factor 17, height of vermillion upper lip	European	[165]
	*CRYGGP*	Face	Cheek morphology partial-least-square model	East Asian +Admixed Ancestry	[168]
	*CRYGGP*	Eye	Factor 8, orbital inclination due to vertical and horizontal position of exocanthion	European	[165]
3	*DLGAP1*	Eye	Upper eyelid sagging severity	European	[170]
		Eye	Upper eyelid sagging severity	European	[170]
		Mouth	Factor 6, height of vermillion Lower lip	European	[165]
3	*MAGEF1, EPHB3*	Eye	Upper eyelid sagging severity	European	[170]
		Nose	Segment 5	European	[169]
		Nose	Nose size	European	[162]
3	*SMG6*	Forehead	Forehead protrusion 1	Hispanic/Latin American	[161]
		Forehead	(Upper forehead slant angle)	East Asian	[146]
		Chin/Lower face	Segment 51	European	[169]
3	*THSD4*	Eye	Right eye tail length	East Asian	[146]
		Eye	Outercanthal width	East Asian	[146]
		Chin/Lower face	Segment 24	European	[169]
5	*Y_RNA*	Face	Factor 13, vertical position of alar curvature relative to upper lip	European	[165]
	*Y_RNA, ARHGAP15*	Chin/Lower face	Chin dimples	European	[162]
	*Y_RNA, CFAP20*	Eye	Factor 14, intercanthal width	European	[165]
	*Y_RNA, MED13*	Face	Factor 4, facial height related to vertical position of gnathion	European	[165]
	*Y_RNA, RPL35AP3*	Mouth	Factor 6, height of vermillion Lower lip	European	[165]
5	*SFRP2, DCHS2*	Nose	Nose roundness 1	Hispanic/Latin American	[161]
			Nose roundness 3	Hispanic/Latin American	[161]
			Nostril size	Hispanic/Latin American	[161]
			Segment 27	African	[163]
5	*SLC24A2, MLLT3*	Nose	Segment 48	African	[163]
	*SLC24A5*	Nose	Nose roundness 3	Hispanic/Latin American	[161]
	*SLC24A5*	Mouth	Lip thickness 1	Hispanic/Latin American	[161]
	*SLC24A5*	Mouth	Lower lip thickness 1	Hispanic/Latin American	[161]
	*SLC24A5*	Mouth	Lower lip thickness 2	Hispanic/Latin American	[161]
5	*SUPT3H*	Forehead	Forehead protrusion 1	Hispanic/Latin American	[161]
	*SUPT3H*	Nose	Nose morphology measurement	East Asian	[160]
	*SUPT3H*	Nose	Nose morphology measurement	East Asian	[160]
	*SUPT3H*	Chin/Lower face	Chin dimples	European	[162]
	*SUPT3H, CDC5L*	Nose	Segment 23	European	[169]
6	*CRB1*	Face	Factor 4, facial height related to vertical position of gnathion	European	[165]
		Mouth	Lip protrusion	Hispanic/Latin American	[161]
		Mouth	Lower lip protrusion	Hispanic/Latin American	[161]
		Chin/Lower face	Chin protrusion 1	Hispanic/Latin American	[161]
		Chin/Lower face	Chin protrusion 2	Hispanic/Latin American	[161]
		Chin/Lower face	Chin dimples	European	[162]
6	*GCC2*	Mouth	Lip protrusion	Hispanic/Latin American	[161]
		Mouth	Lower lip protrusion	Hispanic/Latin American	[161]
		Chin/Lower face	Jaw protrusion 2	Hispanic/Latin American	[161]
		Chin/Lower face	Jaw protrusion 5	Hispanic/Latin American	[161]
		Chin/Lower face	Jaw slope 2	Hispanic/Latin American	[161]
		Chin/Lower face	Lower face flatness	Hispanic/Latin American	[161]
7	*CASC17*	Nose	Columella inclination	Hispanic/Latin American	[161]
		Nose	Nose roundness 1	Hispanic/Latin American	[161]
		Nose	Nose size	Hispanic/Latin American	[161]
		Nose	Segment 5	European	[169]
		Nose	Nasal tip protrusion	East Asian	[146]
		Nose	Profile nasal area	East Asian	[146]
		Chin/Lower face	Chin dimples	European	[162]
8	*ROCR*	Nose	Profile nasal angle	East Asian	[146]
	*ROCR*	Nose	Profile nasal angle	East Asian	[146]
	*ROCR*	Nose	Nasal tip protrusion	East Asian	[146]
	*ROCR*	Nose	Nasolabial angle	East Asian	[146]
	*ROCR*	Nose	Nasal tip protrusion	East Asian	[146]
	*ROCR*	Nose	Nasolabial angle	East Asian	[146]
	*ROCR*	Nose	Nose size	European	[162]
	*ROCR, LINC01152*	Nose	Segment 44	European	[169]
9	*MTX2, RPSAP25*	Eye	Right eye tail length	East Asian	[146]
		Eye	Eye morphology	East Asian	[160]
		Eye	Tangent line angle of er4	East Asian	[146]
		Eye	Right eyelid peak position ratio	East Asian	[146]
		Eye	Tangent line angle of el3	East Asian	[146]
		Eye	Tangent line angle of el4	East Asian	[146]
		Eye	Tangent line angle of el6	East Asian	[146]
		Eye	Tangent line angle of er3	East Asian	[146]
		Mouth	Mouth morphology	East Asian	[160]

## Data Availability

The data presented in this review are available within the article and in the supplementary material.

## Supplementary Materials

The following supporting information can be downloaded at: https://www.mdpi.com/article/10.3390/genes14010136/s1: Table S1: Summary of data obtained from NHGRI-EBI GWAS Catalog for SNPs that met the genome-wide significance *p*-value threshold of (5 × 10^−8^) for the face region, which include traits affecting multiple face areas. RAF = risk allele frequency. OR = Odd ratio. CI = Confidence interval. NR: Not reported [154,165,168]. Table S2: Summary of data obtained from NHGRI-EBI GWAS Catalog for SNPs that met the genome-wide significance *p*-value threshold of (5 × 10^−8^) for traits in the forehead region. RAF = risk allele frequency. OR = Odd ratio. CI = Confidence interval. NR: Not reported [146,161,163]. Table S3: Summary of data obtained from NHGRI-EBI GWAS Catalog for SNPs that met the genome-wide significance p-value threshold of (5 × 10^−8^) for traits in the eye region. RAF = risk allele frequency. OR = Odd ratio. CI = Confidence interval. NR: Not reported [144,145,146,147,148,149,150,151,152,153,154,155,156,157,158,159,160,161,163,168]. Table S4: Summary of data obtained from NHGRI-EBI GWAS Catalog for SNPs that met the genome-wide significance p-value threshold of (5 × 10^−8^) for traits in the nose region. RAF = risk allele frequency. OR = Odd ratio. CI = Confidence interval. NR: Not reported [146,158,160,161,162,163,165,168,169]. Table S5: Summary of data obtained from NHGRI-EBI GWAS Catalog for SNPs that met the genome-wide significance p-value threshold of (5 × 10^−8^) for traits in the mouth region. RAF = risk allele frequency. OR = Odd ratio. CI = Confidence interval. NR: Not reported [146,154,156,160,161,163,165,168,169]. Table S6: Summary of data obtained from NHGRI-EBI GWAS Catalog for SNPs that met the genome-wide significance p-value threshold of (5 × 10^−8^) for traits in the chin/lower face region. RAF = risk allele frequency. OR = Odd ratio. CI = Confidence interval. NR: Not reported [158,160,161,162,163,169].
