# Supplementary material for: Advancement in Human Face Prediction Using DNA
Source: Genes (Basel). 2023 Jan 3;14(1):136. doi: 10.3390/genes14010136 (PMC9858985; doi:10.3390/genes14010136)
Supplement: Supplementary file 1 [file genes-14-00136-s001.zip › genes-2025099-supplementary.pdf]

Title: Advancement in Human Face Prediction Using DNA

Supplementary Materials:

Table S1: Summary of data obtained from NHGRI-EBI GWAS Catalog for SNPs that met the genome-wide significance *p*-value threshold of (5×10^{-8}) for the face region, which include traits affecting multiple face areas. RAF = risk allele frequency. OR = Odd ratio. CI = Confidence interval. NR: Not reported [168], [165], [154].

Reported Trait	Variant and risk allele	Mapped gene	Location	Minor Allele	RAF	OR	Beta	CI	P-value	Reference
Facial width measurement	rs118034464	*LINC02151*	11:116484935	C	0.01	NR	0.7961 unit increase	[0.47-1.12]	3.00E-17	[165]
	rs137985742	*TUBA1C*	12:49226772	C	0.31	NR	0.1529 unit increase	[0.088-0.218]	4.00E-17	[165]
	rs8003054	*OSGEP, KLHL33*	14:20439279	G	0.36	NR	0.1612 unit increase	[0.1-0.22]	6.00E-17	[165]
	rs17228178	*TRPM1, LINC02352*	15:31177725	T	0.28	NR	0.1619 unit increase	[0.097-0.227]	7.00E-17	[165]
	rs11073464	*NR2F2-AS1*	15:96128740	G	0.30	NR	0.1736 unit decrease	[0.11-0.24]	7.00E-17	[165]
	rs148442614	*RPS9, TSEN34*	19:54196103	T	0.01	NR	0.67 unit decrease	[0.39-0.95]	1.00E-16	[165]
	rs374193423	*LINC00607*	2:215658950	C	0.30	NR	0.1794 unit increase	[0.11-0.24]	1.00E-16	[165]
	rs1108585	*PLXND1*	3:129601685	A	0.09	NR	0.2362 unit increase	[0.14-0.34]	5.00E-16	[165]
	rs62328032	*NR*	4:156411459	T	0.05	NR	0.3212 unit decrease	[0.19-0.46]	1.00E-15	[165]
	rs66754540	*DCTD, TENM3*	4:182826850	A	0.11	NR	0.2291 unit increase	[0.13-0.33]	2.00E-15	[165]
	rs6880635	*RASGRF2*	5:80978080	T	0.01	NR	0.8464 unit decrease	[0.49-1.21]	2.00E-15	[165]
	rs181878063	*LINC01470, PRKAA1*	5:152572320	C	0.06	NR	0.3102 unit increase	[0.18-0.44]	2.00E-15	[165]
	rs6579967	*LINC01470*	5:152785450	G	0.06	NR	0.2979 unit increase	[0.18-0.42]	3.00E-15	[165]
	rs58248207	*DNAJC5B*	8:65987353	AT	0.15	NR	0.2018 unit increase	[0.12-0.29]	3.00E-15	[165]
	rs6064603	*LINC01742*	20:57983390	A	0.02	NR	0.5209 unit decrease	[0.3-0.74]	3.00E-15	[165]
	rs4440965	*LINC01435*	10:107761992	G	0.43	NR	0.1443 unit increase	[0.084-0.205]	3.00E-15	[165]
	rs2971996	*DISP1*	1:222936592	G	0.16	NR	0.1896 unit decrease	[0.11-0.27]	4.00E-15	[165]
	rs373711932	*CDK13, RN7SL496P*	7:39928927	C	0.42	NR	0.1413 unit decrease	[0.082-0.2]	4.00E-15	[165]
	rs5767180	*TAFA5*	22:48513656	A	0.01	NR	0.7425 unit increase	[0.45-1.03]	7.00E-15	[165]
	rs79756450	*LINC02683, SPON1*	11:13953912	C	0.04	NR	0.3914 unit increase	[0.24-0.54]	8.00E-15	[165]
	rs1853554	*KRT18P14, TRIM44*	11:35842422	A	0.23	NR	0.1711 unit decrease	[0.099-0.243]	9.00E-15	[165]
	9:90659999	*NR*	Mapping not available	C	0.03	NR	0.4114 unit decrease	[0.24-0.58]	1.00E-14	[165]
Cranial base width	rs117137044	*RNU6-273P, MIPOL1*	14:37190997	A	0.11	NR	1.058 unit decrease	[0.66-1.46]	1.00E-14	[154]
	rs55995849	*MIPOL1*	14:37313885	A	0.11	NR	1.083 unit decrease	[0.68-1.48]	1.00E-14	[154]

	rs17106852	*FOXA1, MIPOL1*	14:37569263	G	0.11	NR	1.104 unit decrease	[0.7-1.51]	1.00E-14	[154]
	rs6129564	*NR*	20:40275563	A	0.12	NR	1.21 unit decrease	[0.83-1.59]	1.00E-14	[154]
Factor 4, facial height related to vertical position of gnathion	rs74513917	*NR*	14:42455609	T	0.01	NR	0.7445 unit increase	[0.46-1.03]	1.00E-12	[165]
	rs7432532	*SCN5A*	3:38587170	C	0.10	NR	0.2484 unit increase	[0.15-0.35]	1.00E-12	[165]
	rs140662893	*GSK3B*	3:119861520	C	0.01	NR	0.7781 unit decrease	[0.45-1.1]	1.00E-12	[165]
	rs73249112	*SLC7A5, KLHDC4*	16:87794485	T	0.04	NR	0.3556 unit decrease	[0.21-0.5]	2.00E-12	[165]
	rs79032298	*MEF2C, MEF2C-AS2*	5:88741557	A	0.02	NR	0.4646 unit decrease	[0.28-0.65]	2.00E-12	[165]
	rs181974282	*NR*	5:155134628	G	0.01	NR	0.7107 unit increase	[0.42-1]	2.00E-12	[165]
	rs76643455	*TMEM132E*	17:34665500	T	0.06	NR	0.301 unit decrease	[0.18-0.43]	2.00E-12	[165]
	rs148770887	*Y_RNA, MED13*	17:62111981	A	0.02	NR	0.5393 unit decrease	[0.31-0.76]	2.00E-12	[165]
	17:60407631	*NR*	Mapping not available	C	0.01	NR	0.6929 unit decrease	[0.43-0.95]	3.00E-12	[165]
	18:48657066	*NR*	Mapping not available	A	0.12	NR	0.2195 unit decrease	[0.13-0.31]	3.00E-12	[165]
	rs148380447	*TERF1, KCNB2*	8:72951519	C	0.01	NR	0.8221 unit decrease	[0.49-1.16]	3.00E-12	[165]
	rs118125078	*RFX3*	9:3227730	T	0.04	NR	0.381 unit decrease	[0.22-0.54]	4.00E-12	[165]
	rs7074498	*FAM241B, MTND1P20*	10:69615672	T	0.01	NR	0.7278 unit increase	[0.43-1.03]	4.00E-12	[165]
	rs59365945	*DENND1B*	1:197738021	GCCT	0.25	NR	0.1765 unit increase	[0.25-0.11]	4.00E-12	[165]
	rs149262213	*SBSPON*	8:73128248	T	0.01	NR	0.8337 unit increase	[1.18-0.49]	5.00E-12	[165]
	rs34949598	*CRB1*	1:197433061	A	0.22	NR	0.173 unit increase	[0.1-0.25]	5.00E-12	[165]
	rs114959389	*RAB3GAP2*	1:220208415	C	0.01	NR	0.7393 unit increase	[0.44-1.04]	5.00E-12	[165]
	rs55721405	*RPS6P23, DOCK9-DT*	13:99136174	T	0.02	NR	0.5292 unit increase	[0.31-0.75]	6.00E-12	[165]
	rs58783610	*NR*	2:57606812	C	0.21	NR	0.1734 unit decrease	[0.1-0.25]	6.00E-12	[165]
	rs12465185	*NR*	2:59860787	G	0.07	NR	0.3052 unit decrease	[0.19-0.42]	6.00E-12	[165]
Factor 9, facial height related to vertical position of nasion	rs150688177	*NR*	13:55478374	G	0.01	NR	0.6895 unit decrease	[0.4-0.97]	1.00E-13	[165]
	rs192381057	*DAOA-AS1*	13:105498801	A	0.01	NR	0.8382 unit increase	[0.49-1.19]	1.00E-13	[165]
	rs112511609	*CCDC33*	15:74226743	A	0.02	NR	0.5299 unit increase	[0.3-0.75]	1.00E-13	[165]
	rs62096916	*HMGN1P31*	18:60776897	A	0.10	NR	0.2395 unit increase	[0.14-0.34]	1.00E-13	[165]
	rs191281700	*ZNF28*	19:52828179	A	0.01	NR	0.8081 unit increase	[0.47-1.15]	1.00E-13	[165]
	rs75352580	*ADGRL4*	1:79090306	A	0.03	NR	0.4347 unit increase	[0.26-0.61]	2.00E-13	[165]
	rs340502	*LINC01833, CAMKMT*	2:44853940	A	0.34	NR	0.1514 unit decrease	[0.089-0.214]	2.00E-13	[165]

	exm246260	*NR*	Mapping not available	G	0.02	NR	0.5866 unit increase	[0.37-0.81]	2.00E-13	[165]
	rs144308062	*TTN, TTN-AS1*	2:178737681	C	0.02	NR	0.5503 unit increase	[0.34-0.76]	3.00E-13	[165]
	rs79124480	*TENM3*	4:182345662	G	0.01	NR	0.7048 unit decrease	[0.4-1.01]	3.00E-13	[165]
	rs7378943	*KIAA0825*	5:94212587	G	0.09	NR	0.2579 unit decrease	[0.15-0.36]	3.00E-13	[165]
	rs6580110	*MIR1303, Y_RNA*	5:154697614	G	0.32	NR	0.1528 unit decrease	[0.088-0.217]	4.00E-13	[165]
	rs9456748	*PRKN*	6:162168986	G	0.44	NR	0.1651 unit increase	[0.11-0.22]	6.00E-13	[165]
	rs10253066	*TAS2R2P, SCIN*	7:12535647	C	0.47	NR	0.1428 unit decrease	[0.083-0.203]	6.00E-13	[165]
	rs10959228	*RPS27AP14, DMRT2*	9:1077149	A	0.35	NR	0.1567 unit increase	[0.094-0.219]	7.00E-13	[165]
	rs10965534	*CCDC187*	9:22953630	T	0.03	NR	0.4161 unit decrease	[0.25-0.59]	7.00E-13	[165]
	rs143168415	*NR*	21:16878888	A	0.01	NR	0.8276 unit decrease	[0.47-1.18]	8.00E-13	[165]
	rs73209858	*BRWD1*	21:39285897	C	0.01	NR	0.7312 unit increase	[0.42-1.04]	9.00E-13	[165]
	rs7917878	*LINC02647*	10:85394782	T	0.11	NR	0.2358 unit increase	[0.14-0.33]	1.00E-12	[165]
Lower facial height	rs17649135	*RPL31P53*	13:57420576	A	0.03	NR	1.428 unit increase	[0.82-2.03]	9.00E-14	[154]
	rs68151129	*GPC6*	13:94331191	T	0.12	NR	0.7802 unit increase	[0.47-1.09]	9.00E-14	[154]
	rs58761416	*LINC02834*	9:86757154	CT	0.24	NR	1.34 unit increase	[0.74-1.94]	1.00E-29	[154]
	rs227726	*C17orf67*	17:56700224	T	0.34	NR	1.242 unit decrease	[0.7-1.79]	3.00E-29	[154]
Upper facial depth	chr1:100612063	*NR*	Mapping not available	C	0.06	NR	1.82 unit decrease	[0.94-2.7]	3.00E-36	[154]
	rs12786942	*TRPC6*	11:101524034	T	0.12	NR	1.429 unit decrease	[0.81-2.05]	2.00E-34	[154]
Middle facial depth	rs12786942	*TRPC6*	11:101524034	T	0.12	NR	1.512 unit decrease	[0.87-2.15]	8.00E-34	[154]
	rs17228178	*TRPM1, LINC02352*	15:31177725	T	0.28	NR	1.215 unit increase	[0.76-1.67]	6.00E-32	[154]
	rs6005551	*MN1*	22:27657842	T	0.23	NR	0.9915 unit decrease	[0.5-1.48]	8.00E-30	[154]
Factor 13, vertical position of alar curvature relative to upper lip	rs35474939	*KCNQ1*	11:2503729	T	0.13	NR	0.2202 unit decrease	[0.13-0.31]	8.00E-74	[165]
	rs10893109	*OR8D4, OR4D5*	11:123933712	C	0.50	NR	0.1449 unit increase	[0.085-0.205]	5.00E-68	[165]
	rs118081607	*LINC02873*	11:130700896	T	0.01	NR	0.8048 unit increase	[0.48-1.13]	6.00E-65	[165]
	rs190597399	*Y_RNA*	X:144191136	A	0.02	NR	0.4133 unit decrease	[0.24-0.59]	7.00E-62	[165]
	rs80062874	*ANO4*	12:100745094	T	0.02	NR	0.4987 unit increase	[0.29-0.7]	8.00E-58	[165]
	rs199814749	*ZRANB2-AS2*	1:71125502	CT	0.09	NR	0.2421 unit decrease	[0.14-0.34]	5.00E-56	[165]
	rs116133561	*ZRANB2-AS2*	1:71353465	G	0.06	NR	0.3097 unit decrease	[0.19-0.43]	2.00E-51	[165]
	rs5778766	*PRDX6-AS1*	1:173458993	T	0.32	NR	0.1582 unit increase	[0.096-0.221]	9.00E-49	[165]
	rs76210638	*RFC3*	13:33928046	T	0.09	NR	0.2521 unit decrease	[0.15-0.36]	1.00E-46	[165]
	rs17124581	*SPATA7, KCNK10*	14:88360338	C	0.06	NR	0.3132 unit increase	[0.19-0.44]	1.00E-46	[165]

	rs145969980	*TTLL13P, GDPGP1*	15:90246301	G	0.01	NR	0.5928 unit increase	[0.35-0.168]	3.00E-46	[165]
	rs117548309	*SUGCT*	7:40703303	G	0.06	NR	0.3102 unit increase	[0.18-0.44]	6.00E-45	[165]
	rs60941879	*FBN3*	19:8069145	T	0.09	NR	0.252 unit increase	[0.15-0.36]	6.00E-45	[165]
	rs144211280	*MEIS3*	19:47410926	T	0.01	NR	0.8524 unit increase	[0.49-1.22]	1.00E-38	[165]
	rs6104460	*CD40, RPL13P2*	20:46100770	T	0.05	NR	0.3145 unit increase	[0.19-0.44]	4.00E-37	[165]
	rs72284640	*DNA2*	10:68441336	T	0.12	NR	0.2297 unit increase	[0.14-0.32]	4.00E-37	[165]
Cheek morphology partial least square model	rs17868256	*CRYGGP*	2:51805635	NR	NR	NR	0.0522 unit increase	NR	8.00E-14	[168]

Table S2. Summary of data obtained from NHGRI-EBI GWAS Catalog for SNPs that met the genome-wide significance *p*-value threshold of (5×10^{-8}) for traits in the forehead region. RAF = risk allele frequency. OR = Odd ratio. CI = Confidence interval. NR: Not reported [146], [161], [163].

Reported Trait	Variant and risk allele	Mapped gene	Location	Minor Allele	RAF	OR	Beta	CI	P-value	Reference
Forehead protrusion 1	rs10948192	*SUPT3H*	6:44979584	NR	NR	NR	NR	NR	2.00E-14	[161]
	rs7966340	*HMGA2*	12:65937585	NR	NR	NR	NR	NR	2.00E-14	[161]
	rs8069947	*SMG6*	17:2082549	NR	NR	NR	NR	NR	3.00E-14	[161]
segment 40	rs77926594	*CDH7*	18:65799204	NR	NR	NR	NR	NR	7.00E-14	[163]
segment 41	rs58409393	*ADAM15*	1:155052831	NR	NR	NR	NR	NR	7.00E-14	[163]
Upper forehead slant angle	rs216215	*SMG6*	17:2237374	T	0.42	NR	0.723 unit decrease	NR	4.00E-14	[146]
	rs363444	*BACH1, GRIK1*	21:29614128	A	0.19	NR	0.05 unit increase	NR	5.00E-14	[146]
Metopion eminence depth	rs3758477	*FAM245A, MIR4678*	10:87503615	T	0.35	NR	0.058 unit increase	NR	5.00E-14	[146]
	rs4283041	*WNT5B, LINC00942*	12:1528747	T	0.17	NR	0.073 unit decrease	NR	6.00E-14	[146]
Forehead height	rs10081631	*RNU6-1035P*	9:81776259	C	0.45	NR	0.676 unit increase	NR	6.00E-14	[146]
Brow ridge height	rs323187	*CPVL*	7:29111091	G	0.42	NR	0.32 unit decrease	NR	6.00E-14	[146]

Table S3. Summary of data obtained from NHGRI-EBI GWAS Catalog for SNPs that met the genome-wide significance *p*-value threshold of (5 × 10^{-8}) for traits in the eye region. RAF = risk allele frequency. OR = Odd ratio. CI = Confidence interval. NR: Not reported [168], [146], [144]–[161], [163]–[154], [155].

Reported trait	Variant and risk allele	Mapped gene	Location	Minor Allele	RAF	OR	Beta	CI	P-value	Reference
Eye position 1	rs17134499	*SGO1P2, RN7SL292P*	7:51764045	NR	NR	NR	NR	NR	5.00E-10	[161]
Factor 14, inter-canthal width	rs139852892	*LINC01643*	22:34269045	A	0.01	NR	0.6596 unit decrease	NR	2.00E-09	[165]
	rs6624621	*PHKA1*	X:72621579	G	0.39	NR	0.1379 unit increase	NR	2.00E-09	[165]
	rs799522	*FRY*	13:31939891	T	0.13	NR	0.2157 unit increase	NR	2.00E-09	[165]
	rs9530186	*MARK2P12*	13:73346327	C	0.24	NR	0.1912 unit decrease	NR	2.00E-09	[165]
	rs877426	*RASA3*	13:114068236	T	0.28	NR	0.1697 unit decrease	NR	2.00E-09	[165]
	rs74019792	*Y_RNA, CFAP20*	16:58108029	A	0.08	NR	0.2801 unit increase	NR	2.00E-09	[165]
	rs72794054	*WWOX*	16:78352046	A	0.04	NR	0.3947 unit decrease	NR	2.00E-09	[165]
	rs56053540	*NR*	16:82376611	G	0.11	NR	0.2166 unit decrease	NR	2.00E-09	[165]
	rs12456112	*GNAL*	18:11872893	G	0.03	NR	0.4391 unit increase	NR	2.00E-09	[165]
	rs3761026	*ARHGAP45*	19:1086044	C	0.47	NR	0.1488 unit decrease	NR	3.00E-09	[165]
	rs1706867	*NR*	2:2517476	G	0.46	NR	0.138 unit increase	NR	3.00E-09	[165]
	rs7673594	*TRIML2, ZFP42*	4:188023576	T	0.19	NR	0.1765 unit decrease	NR	3.00E-09	[165]
	rs5001173	*MAP1B*	5:72159447	A	0.27	NR	0.1566 unit decrease	NR	3.00E-09	[165]
	rs147675704	*NR*	5:121028860	TG	0.24	NR	0.1596 unit decrease	NR	3.00E-09	[165]
	rs148372137	*RAB23, PRIM2*	6:57273975	T	0.01	NR	0.6142 unit decrease	NR	3.00E-09	[165]
	rs17150689	*ATXN7L1*	7:105644232	C	0.01	NR	0.6177 unit increase	NR	3.00E-09	[165]
	rs55940216	*NAT1, ASAH1*	8:18125408	G	0.15	NR	0.2002 unit increase	[0.12-0.28]	3.00E-09	[165]
	rs79687921	*FXN*	9:69053515	G	0.43	NR	0.1435 unit increase	[0.085-0.202]	3.00E-09	[165]
	rs77783563	*PFKP*	10:3116281	A	0.03	NR	0.4409 unit decrease	[0.25-0.63]	3.00E-09	[165]
	rs138166088	*PTER, LINC02654*	10:16388195	C	0.01	NR	0.6929 unit increase	[0.4-0.99]	3.00E-09	[165]
	rs11093404	*PABPC1L2A, PABPC1L2B*	X:73069628	A	0.24	NR	0.1745 unit increase	[0.11-0.23]	4.00E-09	[165]
Factor 2, vertical position of orbits relative to midface	rs16981003	*MYO18B-AS1, MYO18B*	22:25899066	T	0.03	NR	0.3912 unit increase	[0.23-0.55]	8.00E-10	[165]
	rs9615838	*MIR3201*	22:48238657	T	0.03	NR	0.3856 unit increase	[0.22-0.55]	8.00E-10	[165]
	rs78814167	*CAPRIN2, IPO8*	12:30706035	T	0.02	NR	0.5471 unit decrease	[0.32-0.77]	8.00E-10	[165]

	rs11629709	*LINC00924*	15:95409726	G	0.07	NR	0.2812 unit increase	[0.16-0.4]	8.00E-10	[165]
	rs74431973	*NR*	17:52260888	A	0.01	NR	0.6568 unit decrease	[0.39-0.92]	8.00E-10	[165]
	rs185881420	*MAP2K6, LINC01633*	17:69555786	A	0.01	NR	0.7534 unit decrease	[0.44-1.07]	9.00E-10	[165]
	rs12210772	*RNU6ATAC21P*	6:10270897	A	0.08	NR	0.2579 unit increase	[0.15-0.37]	2.00E-09	[165]
	rs9386051	*PHACTR2, LTV1*	6:143839143	C	0.34	NR	0.154 unit decrease	[0.092-0.216]	2.00E-09	[165]
	rs2727513	*UBE2H, MIR183*	7:129805371	T	0.27	NR	0.1564 unit increase	[0.09-0.223]	2.00E-09	[165]
	rs78305304	*RP1*	8:54611320	G	0.04	NR	0.3536 unit increase	[0.21-0.5]	2.00E-09	[165]
	rs118122713	*FAM110B, C8orf89*	8:58233210	T	0.01	NR	0.8324 unit increase	[0.48-1.18]	2.00E-09	[165]
	rs12003807	*RXRA*	9:134478893	C	0.02	NR	0.5843 unit increase	[0.34-0.82]	2.00E-09	[165]
	rs78643721	*TLX1NB*	10:101093461	T	0.03	NR	0.4196 unit increase	[0.25-0.59]	2.00E-09	[165]
	rs5787602	*YWHAZP5*	10:105699973	T	0.13	NR	0.2312 unit increase	[0.14-0.32]	2.00E-09	[165]
	rs199739975	*FTLP10, TMPRSS11F*	4:68140469	T	0.01	NR	0.6487 unit increase	[0.37-0.92]	2.00E-09	[165]
	rs35404815	*ZFYVE27, SFRP5*	10:97763490	G	0.11	NR	0.2209 unit decrease	[0.13-0.31]	2.00E-09	[165]
Factor 8, orbital inclination due to vertical and horizontal position of exocanthion	rs78754071	*ADAM5*	8:39413302	C	0.04	NR	0.3635 unit increase	[0.22-0.51]	1.00E-09	[165]
	rs113684415	*RASEF*	9:82890407	T	0.02	NR	0.5582 unit increase	[0.33-0.78]	1.00E-09	[165]
	rs12377833	*BRINP1*	9:119355455	G	0.29	NR	0.1565 unit decrease	[0.091-0.222]	1.00E-09	[165]
	rs370286690	*FAM25E, WASHC2C*	10:45793871	C	0.05	NR	0.3486 unit decrease	[0.21-0.49]	1.00E-09	[165]
	rs67832371	*NR*	Mapping not available	CTT	0.27	NR	0.163 unit decrease	[0.095-0.231]	1.00E-09	[165]
	rs1885364	*MN1*	22:27698847	G	0.49	NR	0.1501 unit increase	[0.091-0.209]	1.00E-09	[165]
	rs10459261	*WIF1, LINC02389*	12:64993845	G	0.07	NR	0.2664 unit decrease	[0.15-0.38]	1.00E-09	[165]
	rs9322905	*AKAP6*	14:32569655	C	0.40	NR	0.1526 unit increase	[0.091-0.214]	1.00E-09	[165]
	rs151121230	*CGRRF1*	14:54539032	A	0.02	NR	0.4661 unit increase	[0.27-0.66]	1.00E-09	[165]
	rs60260736	*C17orf67*	17:56701283	A	0.02	NR	0.5718 unit increase	[0.35-0.8]	1.00E-09	[165]
	19:9933973	*NR*	Mapping not available	T	0.01	NR	0.7637 unit decrease	[0.47-1.06]	1.00E-09	[165]
	rs6709347	*CRYGGP*	2:51763821	A	0.16	NR	0.1954 unit increase	[0.11-0.28]	2.00E-09	[165]
	rs186934434	*RN7SKP42, RNU6-989P*	2:187210878	A	0.02	NR	0.5801 unit decrease	[0.34-0.82]	2.00E-09	[165]
	rs182151107	*ULK4*	3:41804959	A	0.01	NR	0.858 unit decrease	[0.5-1.22]	2.00E-09	[165]
	rs12512126	*NR3C2*	4:148285284	G	0.27	NR	0.1616 unit decrease	[0.096-0.227]	2.00E-09	[165]
	rs62367758	*ZNF131*	5:43110564	A	0.19	NR	0.2094 unit decrease	[0.13-0.29]	2.00E-09	[165]
	rs857418	*RNU6-793P*	6:14655800	C	0.02	NR	0.5076 unit increase	[0.29-0.72]	2.00E-09	[165]

	rs151095285	*ABCB4*	7:87475134	G	0.01	NR	0.6942 unit increase	[0.4-0.99]	2.00E-09	[165]
	rs10241381	*SDHAF3, DLX5*	7:97035969	G	0.45	NR	0.1424 unit increase	[0.082-0.202]	2.00E-09	[165]
Segment 14	rs80243479	*TBX3, UBA52P7*	12:114918878	NR	NR	NR	NR	NR	7.00E-09	[163]
Segment 28	rs113199279	*TIFAB, NEUROG1*	5:135470624	NR	NR	NR	NR	NR	7.00E-09	[163]
Segment 59	rs148390647	*CLYBL*	13:99890694	NR	NR	NR	NR	NR	7.00E-09	[163]
Eye length	rs1868752	*MIR100HG*	11:122520734	NR	NR	NR	3.19 unit increase	NR	6.00E-10	[168]
Intercanthal width	rs619686	*GSTM2*	1:109676139	G	0.06	NR	0.7629 unit decrease	[0.44-1.08]	9.00E-10	[154]
	rs61852012	*CDH23*	10:71651356	T	0.13	NR	0.4484 unit decrease	[0.23-0.67]	9.00E-10	[154]
	rs11093404	*PABPC1L2A, PABPC1L2B*	X:73069628	A	0.24	NR	0.4272 unit increase	[0.28-0.57]	1.00E-09	[154]
Outercanthal width	rs201983168	*PRPF3*	1:150342506	TACAC	0.33	NR	0.4915 unit decrease	[0.29-0.69]	1.00E-09	[154]
	rs3828785	*HLA-DQB1*	6:32661130	A	0.03	NR	1.449 unit decrease	[0.91-1.99]	1.00E-09	[154]
Left eye angle of en-ps	rs4742094	*KANK1*	9:560077	G	0.27	NR	0.519 unit increase	NR	4.00E-09	[146]
Left eye angle of ex-ps	rs2972180	*CLN8, DLGAP2*	8:1733567	T	0.18	NR	0.543 unit increase	NR	4.00E-09	[146]
Left eye angle of en-ps-ex	rs6994270	*SAMD12*	8:118336256	G	0.15	NR	1.007 unit decrease	NR	4.00E-09	[146]
	rs6016745	*PTPRT*	20:42321543	A	0.19	NR	0.803 unit decrease	NR	4.00E-09	[146]
Right eye tail length	rs970797	*MTX2, RPSAP25*	2:176247091	A	0.33	NR	0.236 unit increase	NR	4.00E-09	[146]
	rs3736712	*WDR27*	6:169557868	C	0.37	NR	0.293 unit increase	NR	5.00E-09	[146]
	rs8026249	*THSD4*	15:71648279	T	0.34	NR	0.23 unit increase	NR	5.00E-09	[146]
Left palpebral fissure height	rs12675712	*SNTB1*	8:120591250	T	0.16	NR	0.147 unit increase	NR	5.00E-09	[146]
	rs17231256	*RPL23AP12, LINC01700*	21:39076963	T	0.13	NR	0.17 unit decrease	NR	5.00E-09	[146]
Intercanthal width	rs7127228	*LINC02725, DNAJB6P1*	11:128032805	A	0.29	NR	0.301 unit decrease	NR	5.00E-09	[146]
Intercanthal width	rs1879682	*LINC01899*	18:71830005	A	0.47	NR	0.289 unit increase	NR	5.00E-09	[146]
Outercanthal width	rs8026249	*THSD4*	15:71648279	T	0.34	NR	0.525 unit increase	NR	6.00E-09	[146]
Eye morphology	rs12033305	*TBX15, WARS2*	1:119020916	NR	NR	NR	NR	NR	7.00E-09	[160]
	rs12633011	*MRPS22*	3:139220153	NR	NR	NR	NR	NR	7.00E-09	[160]
	rs970797	*MTX2, RPSAP25*	2:176247091	NR	NR	NR	NR	NR	7.00E-09	[160]

Double-edged eyelids	rs12570134	*EMX2OS*	10:117524897	T	0.73	1.41	NR	[1.3-1.54]	7.00E-10	[149]
	rs1415425	*EMX2*	10:117586415	A	0.52	1.23	NR	[1.14-1.33]	7.00E-10	[149]
Tangent line angle of er4	rs970797	*MTX2, RPSAP25*	2:176247091	A	0.33	NR	0.012 unit increase	NR	4.00E-09	[146]
	rs750688	*IQCJ-SCHIP1*	3:159640989	A	0.18	NR	0.013 unit decrease	NR	4.00E-09	[146]
Right eyelid peak position ratio	rs17032657	*RERE*	1:8536683	G	0.33	NR	0.006 unit decrease	NR	4.00E-09	[146]
	rs970797	*MTX2, RPSAP25*	2:176247091	A	0.33	NR	0.007 unit decrease	NR	4.00E-09	[146]
Left eyelid medial slant	rs4405545	*PLCG2*	16:81824567	G	0.09	NR	0.02 unit increase	NR	4.00E-09	[146]
Right eyelid medial slant	rs11209151	*GNG12*	1:67773251	G	0.11	NR	0.017 unit increase	NR	4.00E-09	[146]
Tangent line angle of el2	rs7761214	*MIR548A1HG*	6:18588902	A	0.23	NR	0.01 unit increase	NR	5.00E-09	[146]
Tangent line angle of el3	rs970797	*MTX2, RPSAP25*	2:176247091	A	0.33	NR	0.015 unit increase	NR	6.00E-09	[146]
	rs10750754	*NR*	11:106345783	G	0.36	NR	0.011 unit increase	NR	6.00E-09	[146]
	rs17032657	*RERE*	1:8536683	G	0.33	NR	0.013 unit increase	NR	6.00E-09	[146]
	rs970797	*MTX2, RPSAP25*	2:176247091	A	0.33	NR	0.015 unit increase	NR	6.00E-09	[146]
	rs2252421	*LINC02651, RPL5P26*	10:69768968	G	0.25	NR	0.013 unit decrease	NR	6.00E-09	[146]
Tangent line angle of el4	rs970797	*MTX2, RPSAP25*	2:176247091	A	0.33	NR	0.011 unit increase	NR	6.00E-09	[146]
	rs10821147	*FAM120A*	9:93509085	A	0.50	NR	0.01 unit increase	NR	6.00E-09	[146]
	rs2000737	*OR5BP1P, LRRC55*	11:57119393	C	0.10	NR	0.017 unit increase	NR	6.00E-09	[146]
Tangent line angle of el6	rs970797	*MTX2, RPSAP25*	2:176247091	A	0.33	NR	0.012 unit decrease	NR	6.00E-09	[146]
Upper eyelid sagging severity	rs2270588	*TSPAN8*	12:71132634	C	0.46	NR	0.12 unit decrease	NR	3.00E-22	[155]
	rs7602784	*RNU4-73P, YWHAQ*	2:9709268	A	0.28	NR	0.19 unit increase	NR	3.00E-22	[155]
	rs3769560	*TTC27*	2:32818790	G	0.39	NR	0.14 unit increase	NR	5.00E-22	[155]
	rs1515451	*ZNF385D*	3:21846679	G	0.46	NR	0.12 unit decrease	NR	1.00E-21	[155]
	rs1520818	*SIAH2, ERICH6-AS1*	3:150734758	C	0.12	NR	0.36 unit decrease	NR	3.00E-21	[155]
	rs4704521	*LHFPL2, ARSB*	5:78770808	T	0.35	NR	0.15 unit decrease	NR	4.00E-21	[155]
	rs7217473	*CCL15-CCL14*	17:35992028	T	0.05	NR	0.85 unit increase	NR	9.00E-21	[155]
	rs11876749	*DLGAP1*	18:3942902	C	0.47	NR	0.14 unit decrease	NR	1.00E-20	[155]
	rs10499596	*SFRP4*	7:38018725	C	0.09	NR	0.58 unit increase	NR	2.00E-20	[155]
	rs11808632	*SMYD3*	1:245831570	T	0.29	NR	0.07 unit increase	NR	5.00E-20	[155]
	rs6432188	*LINC00570, PPIAP60*	2:11358906	T	0.18	NR	0.09 unit increase	NR	7.00E-20	[155]

	rs6714226	*RPSAP25, HOXD1*	2:176212162	T	0.45	NR	0.07 unit increase	NR	2.00E-19	[155]
	rs6447160	*ATP8A1*	4:42450204	C	0.06	NR	0.15 unit decrease	NR	2.00E-19	[155]
	rs16854624	*ATP8A1*	4:42627021	T	0.07	NR	0.14 unit decrease	NR	3.00E-19	[155]
	rs10077083	*PJA2, CTNND2*	5:109306871	C	0.03	NR	0.23 unit increase	NR	4.00E-19	[155]
	rs12451218	*COX10-AS1*	17:13945915	C	0.41	NR	0.07 unit decrease	NR	4.00E-19	[155]
	rs674017	*TMEM135*	11:87284616	C	0.06	NR	0.15 unit increase	NR	6.00E-19	[155]
	rs17465658	*LINC01362*	1:83024478	C	0.22	NR	0.1 unit decrease	NR	6.00E-19	[155]
	rs1356100	*ZNF385D*	3:21846985	A	0.49	NR	0.11 unit increase	NR	7.00E-19	[155]
	rs4074869	*MAGEF1, EPHB3*	3:184613454	G	0.49	NR	0.12 unit decrease	NR	9.00E-19	[155]
	rs4706522	*KCNQ5*	6:73020894	A	0.12	NR	0.11 unit increase	NR	4.00E-18	[155]
	rs7239297	*DLGAP1*	18:3953220	G	0.49	NR	0.12 unit increase	NR	4.00E-18	[155]
	rs16927253	*MACROH2A2*	10:70088678	NR	NR	4	NR	[2.56-6.25]	1.00E-17	[144]
	rs12599182	*ADAMTS18*	16:77422347	NR	NR	2.27	NR	[1.64-3.03]	2.00E-17	[144]
	rs4109293	*LINC02636, COL13A1*	10:69973565	NR	NR	2.38	NR	[1.69-3.33]	2.00E-17	[144]
	rs174213	*ZFP36L1, MAGOH3P*	14:68843795	T	0.33	1.6	NR	[1.32-1.93]	2.00E-17	[144]

Table S4. Summary of data obtained from NHGRI-EBI GWAS Catalog for SNPs that met the genome-wide significance *p*-value threshold of (5 × 10-8) for traits in the nose region. RAF = risk allele frequency. OR = Odd ratio. CI = Confidence interval. NR: Not reported [168], [146], [158], [160]–[163], [165], [169].

Reported trait	Variant and risk allele	Mapped gene	Location	Minor Allele	RAF	OR	Beta	CI	P-value	Reference
Columella inclination	rs12410669	*PAX7*	1:18663724	NR	NR	NR	NR	NR	4.00E-09	[161]
	chr8:101737776	*NR*	Mapping not available	NR	NR	NR	NR	NR	1.00E-08	[161]
	rs1178103	*HDAC9*	7:18701744	NR	NR	NR	NR	NR	3.00E-08	[161]
	rs11653132	*CASC17*	17:71133691	NR	NR	NR	NR	NR	3.00E-08	[161]
	rs2045323	*NR*	4:153910747	A	NR	NR	0.018 unit increase	NR	3.00E-09	[161]
Columella size	rs10176525	*RPL23AP28, PAX3*	2:222174333	NR	NR	NR	NR	NR	1.00E-11	[161]
	rs75976055	*VPS13B*	8:99614274	NR	NR	NR	NR	NR	4.00E-11	[161]
	rs10810682	*RN7SL720P, BNC2*	9:16929844	NR	NR	NR	NR	NR	8.00E-09	[161]
	rs921120	*LINC01121, SIX2*	2:45045902	NR	NR	NR	NR	NR	1.00E-08	[161]
Factor 11, projection of the nose	rs3923268	*ADAMTS10*	19:8590032	A	0.10	NR	0.2867 unit increase	[0.18-0.39]	5.00E-08	[165]
Factor 21, depth of nasal alae	rs113036800	*NR*	8:57536468	T	0.01	NR	0.6913 unit increase	[0.45-0.93]	1.00E-08	[165]
Factor 7, width of cartilaginous portion	rs62031988	*C16orf82*	16:27006174	C	0.01	NR	0.7004 unit increase	[0.46-0.94]	2.00E-08	[165]
Nose protrusion	rs12427884	*LINC00399, LINC00676*	13:109711188	NR	NR	NR	NR	NR	2.00E-09	[161]
Nose roundness 1	rs9995821	*SFRP2, DCHS2*	4:153907214	NR	NR	NR	NR	NR	2.00E-12	[161]
	rs36192537	*PRDM16*	1:3336678	NR	NR	NR	NR	NR	3.00E-11	[161]
	rs11653132	*CASC17*	17:71133691	NR	NR	NR	NR	NR	2.00E-10	[161]
	rs7633584	*IGSF11*	3:118937668	NR	NR	NR	NR	NR	2.00E-08	[161]
	rs3118389	*RPE65, ELOCP18*	1:68395574	NR	NR	NR	NR	NR	5.00E-08	[161]
Nose roundness 3	rs9995821	*SFRP2, DCHS2*	4:153907214	NR	NR	NR	NR	NR	2.00E-10	[161]
	rs1426654	*SLC24A5*	15:48134287	NR	NR	NR	NR	NR	2.00E-08	[161]
Nose size	rs11653132	*CASC17*	17:71133691	NR	NR	NR	NR	NR	4.00E-12	[161]

	rs12427884	*LINC00399, LINC00676*	13:109711188	NR	NR	NR	NR	NR	4.00E-10	[161]
	rs36192537	*PRDM16*	1:3336678	NR	NR	NR	NR	NR	9.00E-09	[161]
Nostril size	rs9995821	*SFRP2, DCHS2*	4:153907214	NR	NR	NR	NR	NR	2.00E-09	[161]
	rs927833	*LINC01432*	20:22060939	NR	NR	NR	NR	NR	1.00E-08	[161]
Segment 10	rs4912082	*CAPZB*	1:19390302	C	0.30	NR	NR	NR	3.00E-08	[169]
	rs10089785	*TMEM74, EMC2*	8:108587740	C	0.38	NR	NR	NR	3.00E-08	[169]
Segment 11	rs1370926	*PAX3, RPL23AP28*	2:222165783	G	0.22	NR	NR	NR	7.00E-62	[169]
	rs4916071	*LINC01748*	1:60554827	A	0.48	NR	NR	NR	2.00E-51	[169]
	rs742071	*PAX7*	1:18653380	T	0.40	NR	NR	NR	1.00E-15	[169]
	rs6047635	*RPL41P1, LINC01582*	20:21800987	T	0.09	NR	NR	NR	2.00E-12	[169]
	rs634687	*ZNF407, ZADH2*	18:75195005	G	0.07	NR	NR	NR	3.00E-11	[169]
	rs12535551	*EPS15P1, HMGN1P19*	7:46637772	G	0.42	NR	NR	NR	5.00E-11	[169]
	rs116792	*ANKEF1, PARAL1*	20:9990760	G	0.43	NR	NR	NR	5.00E-11	[169]
	rs2401176	*ADAMTSL3*	15:83909612	G	0.48	NR	NR	NR	9.00E-11	[169]
	rs6923760	*PKHD1*	6:51820430	G	0.48	NR	NR	NR	1.00E-10	[169]
	rs66516258	*LINC00382*	13:79914990	T	0.18	NR	NR	NR	1.00E-09	[169]
	rs16834081	*LINC01778, LAPTM5*	1:30819794	C	0.09	NR	NR	NR	2.00E-09	[169]
	rs12070922	*RPL7P10, RN7SKP247*	1:81243161	A	0.12	NR	NR	NR	4.00E-09	[169]
	rs12878658	*DDHD1-DT, RPS3AP46*	14:53611084	A	0.17	NR	NR	NR	5.00E-09	[169]
	rs4686337	*SRGAP3*	3:9269531	T	0.48	NR	NR	NR	2.00E-08	[169]
	rs10189338	*LINC02898, PKDCC*	2:42011482	C	0.29	NR	NR	NR	2.00E-08	[169]
Segment 20	rs1411551	*LINC00676, LINC00399*	13:109720688	A	0.25	NR	NR	NR	4.00E-12	[169]
	rs1902713	*PTPN20, GDF10*	10:47297284	C	0.37	NR	NR	NR	2.00E-08	[169]
	rs7674010	*LEF1-AS1, RPSAP34*	4:108373433	T	0.29	NR	NR	NR	4.00E-08	[169]
Segment 21	rs1520	*KIF6*	6:39559091	G	0.38	NR	NR	NR	2.00E-22	[169]
	rs6113624	*LINC00261, LINC01427*	20:22305792	A	0.40	NR	NR	NR	7.00E-17	[169]
	rs772154	*NCAPH*	2:96355926	T	0.34	NR	NR	NR	1.00E-08	[169]
	rs73457129	*RUNX2*	6:45606313	A	0.15	NR	NR	NR	5.00E-08	[169]
Segment 22	rs9381923	*PKHD1, FTH1P5*	6:51018173	T	0.26	NR	NR	NR	7.00E-19	[169]

	rs62443772	*GLI3*	7:42092350	A	0.21	NR	NR	NR	5.00E-16	[169]
	rs6715010	*LINC01376*	2:19078027	T	0.47	NR	NR	NR	1.00E-14	[169]
	rs2359442	*RNA5SP455, SYT4*	18:43963972	A	0.50	NR	NR	NR	3.00E-09	[169]
	rs12055796	*RCAN2*	6:46338810	T	0.47	NR	NR	NR	2.00E-08	[169]
Segment 23	rs227832	*SUPT3H, CDC5L*	6:44713520	C	0.26	NR	NR	NR	4.00E-37	[169]
	rs9310211	*FOXP1*	3:71182593	A	0.42	NR	NR	NR	5.00E-22	[169]
	rs4811827	*BMP7*	20:57223184	C	0.43	NR	NR	NR	3.00E-09	[169]
Segment 27	rs9995821	*SFRP2, DCHS2*	4:153907214	?	NR	NR	NR	NR	3.00E-22	[163]
	chr3:127963189	*NR*	Mapping not available	NR	NR	NR	NR	NR	2.00E-11	[163]
Segment 44	rs9302943	*ROCR, LINC01152*	17:72038048	A	0.44	NR	NR	NR	1.00E-46	[169]
	rs145965565	*PTCH1*	9:95511023	G	0.09	NR	NR	NR	1.00E-14	[169]
	rs35395759	*LINC01121, SIX2*	2:45123522	T	0.28	NR	NR	NR	4.00E-10	[169]
Segment 45	rs798682	*CADPS2*	7:122347104	A	0.26	NR	NR	NR	9.00E-24	[169]
	rs11190970	*BTRC*	10:101368575	A	0.20	NR	NR	NR	7.00E-09	[169]
	rs149814396	*SFRP1, RNU6-356P*	8:41229590	T	0.25	NR	NR	NR	3.00E-08	[169]
	rs1536446	*SORBS1*	10:95499803	G	0.41	NR	NR	NR	4.00E-08	[169]
Segment 46	rs11665450	*TCF4, RNA5SP459*	18:55177535	T	0.50	NR	NR	NR	1.00E-10	[169]
Segment 48	rs10122939	*SLC24A2, MLLT3*	9:20300845	NR	NR	NR	NR	NR	3.00E-10	[163]
Segment 5	rs9908442	*CASC17*	17:71141807	A	0.37	NR	NR	NR	3.00E-46	[169]
	rs287104	*KCTD15*	19:33800090	G	0.35	NR	NR	NR	4.00E-37	[169]
	rs58022575	*MAGEF1, EPHB3*	3:184615382	G	0.47	NR	NR	NR	3.00E-26	[169]
	rs2098990	*TBX3-AS1, UBA52P7*	12:114982050	A	0.47	NR	NR	NR	7.00E-25	[169]
	rs9633535	*ARID5B*	10:62076329	T	0.41	NR	NR	NR	4.00E-11	[169]
	rs74921869	*FGFRL1*	4:1019594	A	0.19	NR	NR	NR	4.00E-11	[169]
	rs11842203	*SOX21-AS1, LINC00557*	13:94856400	G	0.05	NR	NR	NR	7.00E-11	[169]
	rs4582322	*PWWP2A*	5:160082393	G	0.40	NR	NR	NR	1.00E-08	[169]
	rs7806852	*DDX43P3, EIF4EP4*	7:81589002	G	0.17	NR	NR	NR	2.00E-08	[169]
Segment 52	rs56063440	*CACNA2D3*	3:54697347	NR	NR	NR	NR	NR	1.00E-08	[163]
Segment 53	chr4:24163580	*NR*	Mapping not available	NR	NR	NR	NR	NR	9.00E-09	[163]
Segment 54	rs16983329	*LINC01432*	20:22054559	NR	NR	NR	NR	NR	2.00E-08	[163]

Nasion position	rs7559271	*PAX3*	2:222203567	G	NR	NR	0.082 unit increase	NR	4.00E-11	[158]
Nose wing breadth	rs17640804	*GLI3*	7:42091791	T	NR	NR	0.00049 NR decrease	NR	5.00E-10	[158]
Nose protrusion	rs2045323	*NR*	4:153910747	A	NR	NR	0.00059 NR decrease	NR	1.00E-08	[158]
Nose tip angle	rs2045323	*NR*	4:153910747	A	NR	NR	0.016 unit increase	NR	2.00E-08	[158]
Nose bridge breadth	rs1852985	*RUNX2, SUPT3H*	6:45361919	T	NR	NR	0.00044 unit increase	NR	2.00E-08	[158]
Nose morphology measurement	rs118078182	*COL23A1*	5:178495197	NR	NR	NR	2.07 unit decrease	NR	3.00E-10	[168]
	rs2045323	*NR*	4:153910747	NR	NR	NR	NR	NR	2.00E-17	[160]
Profile nasal angle	rs2193054	*ROCR*	17:72029668	C	0.47	NR	0.007 unit decrease	[0.0050-0.0090]	6.00E-17	[146]
	rs1859979	*NR*	17:71433002	C	0.46	NR	0.007 unit decrease	[0.005-0.009]	1.00E-14	[146]
	rs2058742	*ROCR*	17:72044406	T	0.28	NR	0.007 unit increase	NR	8.00E-14	[146]
	rs2159042	*NR*	17:71413188	C	0.48	NR	0.006 unit decrease	NR	2.00E-11	[146]
	rs2024070	*NR*	17:71423790	C	0.48	NR	0.006 unit decrease	NR	3.00E-11	[146]
	rs10744843	*NR*	12:114974938	A	0.27	NR	0.006 unit decrease	NR	5.00E-08	[146]
Profile nasal area	rs1859979	*NR*	17:71433002	C	0.46	NR	0.018 unit increase	[0.012-0.024]	7.00E-12	[146]
	rs2159042	*NR*	17:71413188	C	0.48	NR	0.019 unit increase	NR	3.00E-11	[146]
	rs2024070	*NR*	17:71423790	C	0.48	NR	0.019 unit increase	NR	5.00E-11	[146]
	rs9915190	*CASC17*	17:71146487	A	0.45	NR	0.016 unit decrease	[0.01-0.022]	2.00E-09	[146]
Nasolabial angle	rs1859979	*NR*	17:71433002	C	0.46	NR	0.013 unit decrease	[0.0091-0.0169]	1.00E-10	[146]
	rs2193054	*ROCR*	17:72029668	C	0.47	NR	0.012 unit decrease	NR	5.00E-09	[146]
	rs3105176	*VPS13B*	8:99499670	C	0.42	NR	0.013 unit increase	NR	1.00E-08	[146]
	rs2058742	*ROCR*	17:72044406	T	0.28	NR	0.013 unit increase	NR	4.00E-08	[146]
Nasal tip protrusion	rs2058742	*ROCR*	17:72044406	T	0.28	NR	0.02 unit decrease	NR	2.00E-10	[146]
	rs1859979	*NR*	17:71433002	C	0.46	NR	0.017 unit increase	[0.011-0.023]	2.00E-10	[146]
	rs2024070	*NR*	17:71423790	C	0.48	NR	0.018 unit increase	NR	8.00E-10	[146]
	rs2159042	*NR*	17:71413188	C	0.48	NR	0.017 unit increase	NR	1.00E-09	[146]
	rs9915190	*CASC17*	17:71146487	A	0.45	NR	0.016 unit decrease	[0.01-0.022]	2.00E-09	[146]
	rs2193054	*ROCR*	17:72029668	C	0.47	NR	0.017 unit increase	[0.011-0.023]	5.00E-09	[146]
Subnasal width	rs2206437	*RN7SL680P*	20:39364098	A	0.26	NR	0.283 unit decrease	[0.19-0.38]	2.00E-09	[146]

Nasal width	rs2424399	*LINC01726, LINC01727*	20:21651907	C	0.23	NR	0.377 unit increase	[0.24-0.52]	3.00E-08	[154]
Nasal ala length	rs8007643	*RNASE3, RNASE2*	14:20897642	T	0.07	NR	1.064 unit increase	[0.70-1.43]	3.00E-08	[154]
Nose morphology measurement	rs12208924	*RUNX2, SUPT3H*	6:45374314	NR	NR	NR	NR	NR	4.00E-08	[160]
Nasal bridge angle	rs9327968	*NR*	5:106632482	C	0.22	NR	0.52 unit decrease	NR	5.00E-08	[146]
Nose morphology measurement	rs118078182	*COL23A1*	5:178495197	NR	NR	NR	2.07 unit decrease	NR	3.00E-10	[168]
Nose morphology partial least square [illegible]	rs3920540	*LINC01428*	20:7087091	NR	NR	NR	0.0652 unit increase	NR	5.00E-08	[168]
Nose morphology measurement	rs2045323	*NR*	4:153910747	NR	NR	NR	NR	NR	2.00E-17	[160]
Nose morphology measurement	rs11988731	*VPS13B*	8:99066698	NR	NR	NR	NR	NR	6.00E-11	[160]
Nose morphology measurement	rs4714845	*SUPT3H*	6:45262684	NR	NR	NR	NR	NR	6.00E-09	[160]
Nose morphology measurement	rs66604011	*SUPT3H*	6:45085322	NR	NR	NR	NR	NR	1.00E-08	[160]
Nose size	chr3:71227306	*NR*	Mapping not available	NR	NR	NR	0.049 unit increase	[0.040-0.059]	8.00E-26	[162]
	rs13097965	*MAGEF1, EPHB3*	3:184621969	NR	NR	NR	0.033 unit decrease	[0.041-0.025]	2.00E-15	[162]
	rs56063440	*CACNA2D3*	3:54697347	NR	NR	NR	0.03 unit increase	[0.021-0.039]	6.00E-11	[162]
	rs2929451	*PPP1R3B, RNU6-1151P*	8:9227785	NR	NR	NR	0.027 unit increase	[0.019-0.035]	6.00E-11	[162]
	rs5781117	*LYPLAL1-AS1*	1:219468846	NR	NR	NR	0.028 unit increase	[0.020-0.037]	2.00E-10	[162]
	rs146188897	*LINC00676*	13:109720345	NR	NR	NR	0.03 unit increase	[0.021-0.039]	3.00E-10	[162]
	rs10809266	*RPS27AP14, DMRT2*	9:1106093	NR	NR	NR	0.027 unit increase	[0.018-0.036]	1.00E-09	[162]
	rs702489	*GLIS1*	1:53732015	NR	NR	NR	0.038 unit increase	[0.025-0.050]	3.00E-09	[162]
	rs35130793	*BMP7*	20:57217109	NR	NR	NR	0.025 unit increase	[0.017-0.033]	3.00E-09	[162]
	rs2224309	*RPL15P2, LINC02279*	14:94867341	NR	NR	NR	0.031 unit decrease	[0.041-0.021]	4.00E-09	[162]
	rs10761129	*ROR2*	9:91724039	NR	NR	NR	0.026 unit decrease	[0.034-0.017]	7.00E-09	[162]
	chr6:44820741	*NR*	Mapping not available	NR	NR	NR	0.024 unit decrease	[0.032-0.016]	1.00E-08	[162]
	rs34091987	*ROCR*	17:72029446	NR	NR	NR	0.025 unit increase	[0.016-0.034]	3.00E-08	[162]

	rs10779169	*NR*	12:85574026	NR	NR	NR	0.023 unit decrease	[0.031-0.015]	4.00E-08	[162]
	rs424737	*ROBO1*	3:78766756	NR	NR	NR	0.025 unit decrease	[0.033-0.016]	5.00E-08	[162]
	rs34702092	*RAD51B*	14:68525031	NR	NR	NR	0.026 unit decrease	[0.035-0.016]	5.00E-08	[162]

Table S5. Summary of data obtained from NHGRI-EBI GWAS Catalog for SNPs that met the genome-wide significance *p*-value threshold of (5×10^{-8}) for traits in the mouth region. RAF = risk allele frequency. OR = Odd ratio. CI = Confidence interval. NR: Not reported [168], [146], [160], [161], [163], [165], [154], [169], [156].

Reported trait	Variant and risk allele	Mapped gene	Location	Minor Allele	RAF	OR	Beta	CI	P-value	Reference
Mouth morphology measurement	rs4267450	*NR*	19:28336699	A	0.18	NR	0.1839 unit decrease	[0.11-0.26]	7.00E-12	[165]
Mouth morphology measurement	rs2211560	*NFIA*	1:60799963	T	0.45	NR	0.149 unit decrease	[0.09-0.208]	8.00E-12	[165]
Mouth morphology measurement	rs1285495	*LINC01036*	1:187328751	G	0.40	NR	0.144 unit decrease	[0.084-0.204]	1.00E-11	[165]
Mouth morphology measurement	rs4685943	*LINC00620*	3:5493590	T	0.01	NR	0.7797 unit increase	[0.47-1.09]	1.00E-11	[165]
Mouth morphology measurement	rs145030395	*LINC02477*	4:160452045	A	0.01	NR	0.8365 unit increase	[0.48-1.19]	1.00E-11	[165]
Mouth morphology measurement	rs117362612	*RBMXP1*	6:48288656	A	0.01	NR	0.8015 unit increase	[0.47-1.13]	1.00E-11	[165]
Mouth morphology measurement	rs55671240	*TRMT11*	6:126128916	A	0.41	NR	0.1462 unit increase	[0.086-0.206]	2.00E-11	[165]
Mouth morphology measurement	rs2151522	*NR*	6:126888948	A	0.39	NR	0.1547 unit increase	[0.094-0.215]	2.00E-11	[165]
Mouth morphology measurement	rs1178127	*HDAC9*	7:18727720	G	0.26	NR	0.1672 unit increase	[0.1-0.23]	2.00E-11	[165]
Mouth morphology measurement	rs72680930	*OXR1*	8:106504002	G	0.01	NR	0.7774 unit increase	[0.45-1.11]	2.00E-11	[165]
Mouth morphology measurement	rs117477265	*NHLRC2*	10:113865288	T	0.05	NR	0.3472 unit decrease	[0.21-0.49]	2.00E-11	[165]
Mouth morphology measurement	12:128960122	*NR*	Mapping not available	T	0.01	NR	0.9376 unit increase	[0.59-1.29]	2.00E-11	[165]
Mouth morphology measurement	11:63826898	*NR*	Mapping not available	G	0.01	NR	0.7681 unit decrease	[0.44-1.09]	2.00E-11	[165]
Mouth morphology measurement	rs11180096	*ATXN7L3B*	12:74519416	C	0.03	NR	0.449 unit increase	[0.26-0.63]	2.00E-11	[165]
Mouth morphology measurement	rs6538174	*NAV3*	12:77443212	G	0.25	NR	0.1637 unit decrease	[0.095-0.232]	3.00E-11	[165]
Mouth morphology measurement	rs182099288	*POMP*	13:28664099	C	0.01	NR	0.848 unit decrease	[0.49-1.2]	3.00E-11	[165]
Mouth morphology measurement	rs77848072	*LINC00922*	16:65499829	C	0.01	NR	0.8842 unit increase	[0.53-1.24]	3.00E-11	[165]

Mouth morphology measurement	rs118035325	*NFATC1*	18:79400748	T	0.02	NR	0.4972 unit increase	[0.3-0.7]	3.00E-11	[165]
Factor 15, philtrum width	rs188166084	*RNU6-783P*	11:23889343	C	0.48	NR	0.1426 unit increase	[0.083-0.202]	1.00E-08	[165]
	rs9516	*KCNE3*	11:74455233	G	0.44	NR	0.1408 unit decrease	[0.082-0.2]	1.00E-08	[165]
	rs331334	*DMD*	X:32288709	T	0.41	NR	0.1273 unit decrease	[0.076-0.178]	1.00E-08	[165]
	rs143386295	*AIFM1, ELF4*	X:130112644	T	0.01	NR	0.7395 unit decrease	[0.43-1.05]	1.00E-08	[165]
	rs142620191	*TBX5*	12:114370639	CCT	0.20	NR	0.1831 unit increase	[0.11-0.26]	1.00E-08	[165]
	rs12580740	*LINC02439*	12:118839490	T	0.06	NR	0.3095 unit increase	[0.18-0.44]	1.00E-08	[165]
	rs9567488	*NUFIP1, LINC00330*	13:44855298	T	0.22	NR	0.1752 unit increase	[0.1-0.25]	1.00E-08	[165]
	rs34833487	*RNA5SP38*	13:106163884	CT	0.13	NR	0.242 unit increase	[0.15-0.33]	1.00E-08	[165]
	rs75617718	*LINC02134, ABCC12*	16:48060840	T	0.02	NR	0.4864 unit increase	[0.28-0.69]	1.00E-08	[165]
	rs117450953	*LONP2*	16:48258340	T	0.02	NR	0.526 unit increase	[0.31-0.74]	1.00E-08	[165]
	rs139724140	*SIAH1*	16:48367763	T	0.02	NR	0.4757 unit increase	[0.28-0.67]	1.00E-08	[165]
	rs3851779	*NXN*	17:804089	C	0.13	NR	0.2068 unit decrease	[0.12-0.29]	1.00E-08	[165]
	rs145984379	*LINC02775*	1:214143432	T	0.01	NR	0.8007 unit increase	[0.46-1.14]	1.00E-08	[165]
	rs115648813	*LINC01828, LINC01829*	2:67196530	C	0.03	NR	0.4252 unit decrease	[0.25-0.6]	2.00E-08	[165]
	rs113358490	*NPAS2, NPAS2-AS1*	2:100977542	A	0.01	NR	0.7548 unit increase	[0.44-1.07]	2.00E-08	[165]
	rs372574584	*CSRNP3*	2:165666620	A	0.29	NR	0.1564 unit increase	[0.09-0.223]	2.00E-08	[165]
	rs62247992	*POMGNT2*	3:43095204	A	0.16	NR	0.1904 unit increase	[0.11-0.27]	2.00E-08	[165]
	rs62257590	*DNAH1*	3:52334303	G	0.03	NR	0.3817 unit increase	[0.22-0.54]	2.00E-08	[165]
	rs71596245	*SH3D19*	4:151264428	CA	0.47	NR	0.1403 unit decrease	[0.081-0.2]	2.00E-08	[165]
	rs10072935	*NR*	5:68412402	A	0.28	NR	0.1624 unit increase	[0.097-0.228]	2.00E-08	[165]
	rs148586546	*RUFY1, U4*	5:179521364	A	0.08	NR	0.2587 unit increase	[0.15-0.37]	2.00E-08	[165]
	rs140109562	*RNU6-682P, RPL10P19*	8:8949768	G	0.01	NR	0.8405 unit decrease	[0.49-1.19]	2.00E-08	[165]
	rs2379251	*NR*	8:141657166	C	0.22	NR	0.1736 unit decrease	[0.1-0.24]	2.00E-08	[165]
	rs117558151	*GNA14, GNA14-AS1*	9:77502833	C	0.02	NR	0.524 unit increase	[0.32-0.73]	2.00E-08	[165]
	rs6076065	*NAPB*	20:23387774	A	0.40	NR	0.1417 unit decrease	[0.082-0.201]	2.00E-08	[165]
	rs2424533	*NAPB*	20:23395270	T	0.40	NR	0.1435 unit decrease	[0.084-0.203]	2.00E-08	[165]
	rs10159656	*PCDH15*	10:54461990	G	0.01	NR	0.5902 unit decrease	[0.34-0.168]	1.00E-08	[165]

Factor 3, length of philtrum	rs144225767	*BICC1, RN7SKP196*	10:58862443	T	0.24	NR	0.1792 unit increase	[0.11-0.25]	1.00E-08	[165]
	rs201657578	*NR*	2:183457924	GTGCA	0.01	NR	0.8352 unit decrease	[0.50-1.17]	2.00E-08	[165]
	rs201900072	*LINC02098, LINC02725*	11:128190250	G	0.14	NR	0.201 unit decrease	[0.12-0.29]	2.00E-08	[165]
	rs186496085	*NR*	X:113378184	G	0.01	NR	0.713 unit increase	[0.43-1]	2.00E-08	[165]
	rs117438382	*SRRM4*	12:118976759	A	0.01	NR	0.8872 unit increase	[0.57-1.2]	2.00E-08	[165]
	rs1887596	*WASF3, GPR12*	13:26697233	G	0.32	NR	0.1741 unit increase	[0.11-0.24]	2.00E-08	[165]
	rs137862450	*FMN1*	15:32966273	GT	0.05	NR	0.3251 unit increase	[0.19-0.46]	2.00E-08	[165]
	rs192393731	*RGS7*	1:240933088	C	0.03	NR	0.4316 unit increase	[0.25-0.61]	2.00E-08	[165]
	rs34282335	*CNTNAP5*	2:124604230	G	0.14	NR	0.202 unit decrease	[0.12-0.29]	2.00E-08	[165]
	rs11369626	*RAP1BP2*	3:104161514	A	0.05	NR	0.3273 unit increase	[0.19-0.46]	2.00E-08	[165]
	rs12643302	*ADH1B*	4:171407309	C	0.29	NR	0.1538 unit decrease	[0.088-0.22]	2.00E-08	[165]
	rs4958231	*WSPAR*	5:133819778	G	0.37	NR	0.1482 unit increase	[0.087-0.21]	2.00E-08	[165]
	rs62486163	*NR*	7:153748297	A	0.40	NR	0.1377 unit decrease	[0.08-0.196]	2.00E-08	[165]
	rs888349	*KCNK9*	8:139657502	G	0.22	NR	0.1673 unit increase	[0.096-0.239]	2.00E-08	[165]
	rs113978995	*MIR1302-7*	8:141804582	C	0.02	NR	0.5064 unit increase	[0.29-0.72]	2.00E-08	[165]
Factor 17, height of vermillion upper lip	rs146579684	*LINC02206, HMGN2P47*	15:66982554	A	0.01	NR	0.8459 unit decrease	[0.49-1.2]	1.00E-08	[165]
	rs4780490	*LINC01195, RNA5SP403*	16:9537505	C	0.36	NR	0.1498 unit increase	[0.087-0.212]	1.00E-08	[165]
	rs4444359	*RMI2, LITAF*	16:11471156	A	0.20	NR	0.1991 unit decrease	[0.12-0.27]	1.00E-08	[165]
	rs61629263	*CFAP97D1*	17:43782280	G	0.28	NR	0.1554 unit decrease	[0.09-0.22]	1.00E-08	[165]
	rs554111	*KIF17*	1:20715807	C	0.31	NR	0.1642 unit decrease	[0.099-0.229]	1.00E-08	[165]
	2:53550611	*NR*	Mapping not available	T	0.01	NR	0.762 unit decrease	[0.45-1.07]	1.00E-08	[165]
	rs1605857	*PRCPP1*	2:101560771	C	0.40	NR	0.1423 unit decrease	[0.082-0.203]	1.00E-08	[165]
	rs138802801	*CRYGFP, MEAF6P1*	2:209169035	C	0.01	NR	0.9963 unit decrease	[0.63-1.36]	1.00E-08	[165]
	rs148598141	*RPL21P37, MAP2*	2:209352374	G	0.01	NR	0.8378 unit decrease	[0.49-1.19]	1.00E-08	[165]
	rs11898702	*SPAG16*	2:213467791	G	0.02	NR	0.4998 unit increase	[0.29-0.71]	1.00E-08	[165]
	rs9325389	*MUC4, LINC01983*	3:195829508	T	0.29	NR	0.1547 unit increase	[0.089-0.221]	1.00E-08	[165]
	rs143838665	*LINC02224*	5:44518479	CT	0.21	NR	0.1683 unit decrease	[0.097-0.24]	1.00E-08	[165]
	rs62375660	*PPP2R2B, GPR154*	5:146578904	T	0.03	NR	0.3965 unit decrease	[0.23-0.56]	1.00E-08	[165]
	rs144345632	*UFL1-AS1*	6:96439019	TA	0.20	NR	0.1732 unit increase	[0.1-0.25]	1.00E-08	[165]

	rs1845631	*NR*	8:34177997	C	0.26	NR	0.1829 unit decrease	[0.12-0.25]	1.00E-08	[165]
	rs72713618	*FREM1*	9:14883256	A	0.02	NR	0.5917 unit decrease	[0.39-0.8]	1.00E-08	[165]
	rs372248726	*CHRNA4*	20:63369848	T	0.01	NR	0.632 unit increase	[0.37-0.9]	1.00E-08	[165]
	rs116924979	*SH2D4B*	10:80639639	T	0.02	NR	0.5424 unit decrease	[0.31-0.77]	1.00E-08	[165]
	rs186247051	*MYOF*	10:93379825	A	0.01	NR	0.6059 unit decrease	[0.35-0.87]	1.00E-08	[165]
	rs377161956	*LINC02181*	16:87004271	C	0.03	NR	0.4141 unit decrease	[0.24-0.59]	2.00E-08	[165]
	rs1015495	*ANOS1, DRAXINP1*	X:8743713	A	0.34	NR	0.1304 unit decrease	[0.078-0.183]	2.00E-08	[165]
	rs191407208	*HDAC6*	X:48821632	A	0.01	NR	0.8159 unit decrease	[0.48-1.15]	2.00E-08	[165]
	rs187026599	*VDAC1P2, GAGE1*	X:49613888	G	0.01	NR	0.748 unit decrease	[0.43-1.07]	2.00E-08	[165]
	rs113233319	*MRPS36P5*	12:42959610	G	0.02	NR	0.5432 unit decrease	[0.31-0.78]	2.00E-08	[165]
	rs73228881	*OBI1-AS1*	13:78432067	C	0.14	NR	0.2026 unit increase	[0.12-0.29]	2.00E-08	[165]
	rs114351729	*SYNE3, LINC02292*	14:95394460	T	0.02	NR	0.4941 unit increase	[0.29-0.7]	2.00E-08	[165]
Factor 6, height of vermillion lower lip	rs201917376	*AGAP6, TIMM23B-AGAP6*	10:49997992	CAT	0.07	NR	0.3004 unit increase	[0.18-0.42]	2.00E-08	[165]
	rs143569928	*LINC02726*	11:23783102	T	0.01	NR	0.6715 unit decrease	[0.39-0.95]	2.00E-08	[165]
	rs142491398	*DANT2, RN7SL712P*	X:115978123	T	0.05	NR	0.268 unit increase	[0.16-0.38]	2.00E-08	[165]
	rs17139233	*RBFOX1*	16:6047041	G	0.18	NR	0.2001 unit increase	[0.12-0.28]	2.00E-08	[165]
	rs117241547	*XYLT1*	16:17430368	T	0.01	NR	0.7132 unit increase	[0.45-0.98]	2.00E-08	[165]
	rs11643520	*RBBP6*	16:24520397	C	0.11	NR	0.2174 unit increase	[0.12-0.31]	2.00E-08	[165]
	rs139244548	*DLGAP1*	18:3718339	G	0.01	NR	0.7326 unit decrease	[0.42-1.05]	2.00E-08	[165]
	rs77246228	*LINC01781*	1:80596655	G	0.09	NR	0.2556 unit decrease	[0.15-0.36]	2.00E-08	[165]
	rs76876052	*KRT8P45, SMU1P1*	1:157073077	AT	0.23	NR	0.161 unit decrease	[0.092-0.23]	2.00E-08	[165]
	rs1109916	*RN7SL299P*	1:232182198	C	0.30	NR	0.1549 unit decrease	[0.09-0.219]	2.00E-08	[165]
	rs116753621	*RBMS3, RBMS3-AS2*	3:29530073	A	0.02	NR	0.5859 unit increase	[0.35-0.82]	2.00E-08	[165]
	rs7688540	*ZNF732, ZNF876P*	4:267367	G	0.21	NR	0.1771 unit decrease	[0.099-0.256]	2.00E-08	[165]
	rs142229939	*TENM2*	5:167260293	GA	0.01	NR	0.8655 unit increase	[0.49-1.24]	3.00E-08	[165]
	rs79228291	*NR*	Mapping not available	A	0.11	NR	0.2279 unit decrease	[0.13-0.33]	3.00E-08	[165]
	rs145147495	*Y_RNA, RPL35AP3*	6:136968827	A	0.01	NR	0.7289 unit increase	[0.45-1]	3.00E-08	[165]
	rs79577636	*ADAP1, GET4*	7:896555	A	0.01	NR	0.8104 unit increase	[0.48-1.14]	3.00E-08	[165]
	rs17150569	*NXPH1*	7:8527717	T	0.01	NR	0.7558 unit decrease	[0.44-1.07]	3.00E-08	[165]

	rs117584411	*NR*	7:19503337	T	0.03	NR	0.4036 unit increase	[0.23-0.58]	3.00E-08	[165]
	rs73708431	*NR*	7:91489997	G	0.01	NR	0.8936 unit increase	[0.53-1.26]	3.00E-08	[165]
	rs1027479	*TMEM74*	8:108713279	C	0.36	NR	0.1526 unit increase	[0.092-0.213]	3.00E-08	[165]
	rs7278536	*NF1P3*	21:14003186	G	0.39	NR	0.1421 unit increase	[0.083-0.201]	3.00E-08	[165]
	rs72771288	*LINC02660, LINC02639*	10:3873288	T	0.01	NR	0.9495 unit increase	[0.59-1.31]	3.00E-08	[165]
Factor 5, width of mouth relative to central midface	rs7117543	*GALNT18*	11:11466726	C	0.05	NR	0.3448 unit increase	[0.26-0.43]	3.00E-11	[165]
	rs12565500	*ADGRL4*	1:79015968	A	0.16	NR	0.1907 unit decrease	[0.11-0.27]	3.00E-11	[165]
	rs4291556	*LARP7P1, FMOD*	1:203386941	C	0.05	NR	0.3182 unit increase	[0.18-0.45]	3.00E-11	[165]
	rs80107998	*RGS7, FH*	1:241419648	T	0.04	NR	0.356 unit increase	[0.2-0.51]	4.00E-11	[165]
	rs1404872	*SDK1*	7:3305354	A	0.09	NR	0.2705 unit decrease	[0.17-0.37]	4.00E-11	[165]
	rs17133277	*SDK1*	7:3407187	G	0.09	NR	0.2476 unit decrease	[0.14-0.35]	4.00E-11	[165]
	rs4744531	*WDR5-DT, ARF4P1*	9:134119110	A	0.12	NR	0.2327 unit increase	[0.14-0.32]	4.00E-11	[165]
	rs6123683	*BMP7*	20:57249453	G	0.35	NR	0.148 unit increase	[0.086-0.21]	4.00E-11	[165]
	10:116210229	*NR*	Mapping not available	T	0.01	NR	0.718 unit decrease	[0.44-1]	4.00E-11	[165]
	rs2390357	*CLYBL*	13:99803868	G	0.35	NR	0.1529 unit decrease	[0.09-0.215]	5.00E-11	[165]
	rs141606281	*PCCA*	13:100217806	T	0.03	NR	0.4203 unit decrease	[0.25-0.59]	5.00E-11	[165]
	rs75870422	*PCCA*	13:100323837	C	0.03	NR	0.4196 unit decrease	[0.24-0.59]	5.00E-11	[165]
	rs116711337	*CDC42EP3*	2:37678515	C	0.01	NR	0.6365 unit decrease	[0.37-0.91]	5.00E-11	[165]
	rs36106334	*MECOM, LINC01266*	3:935095	G	0.29	NR	0.1536 unit decrease	[0.088-0.219]	5.00E-11	[165]
	rs71288019	*SACM1L*	3:45710501	CT	0.46	NR	0.158 unit increase	[0.099-0.217]	5.00E-11	[165]
	rs6414928	*MSNP1*	5:25907800	A	0.22	NR	0.1868 unit increase	[0.12-0.26]	6.00E-11	[165]
	rs112079675	*HEXB*	5:74648491	C	0.02	NR	0.524 unit increase	[0.31-0.74]	6.00E-11	[165]
Lip protrusion	rs2786116	*CRB1*	1:197364649	NR	NR	NR	NR	NR	9.00E-09	[161]
	rs142166760	*GCC2*	2:108502395	NR	NR	NR	NR	NR	9.00E-09	[161]
	rs6570789	*STXBP5-AS1*	6:147080057	NR	NR	NR	NR	NR	9.00E-09	[161]
Lower lip protrusion	rs2786116	*CRB1*	1:197364649	NR	NR	NR	NR	NR	9.00E-09	[161]
	rs142166760	*GCC2*	2:108502395	NR	NR	NR	NR	NR	9.00E-09	[161]
	rs6570789	*STXBP5-AS1*	6:147080057	NR	NR	NR	NR	NR	9.00E-09	[161]
Lip thickness 1	rs907613	*LSP1*	11:1853062	NR	NR	NR	NR	NR	7.00E-09	[161]
	rs1426654	*SLC24A5*	15:48134287	NR	NR	NR	NR	NR	8.00E-09	[161]

Lower lip thickness 1	rs907613	*LSP1*	11:1853062	NR	NR	NR	NR	NR	8.00E-09	[161]
	rs1426654	*SLC24A5*	15:48134287	NR	NR	NR	NR	NR	8.00E-09	[161]
Lower lip thickness 2	rs3790553	*WARS2*	1:119036653	NR	NR	NR	NR	NR	8.00E-09	[161]
	rs907613	*LSP1*	11:1853062	NR	NR	NR	NR	NR	8.00E-09	[161]
	rs1426654	*SLC24A5*	15:48134287	NR	NR	NR	NR	NR	8.00E-09	[161]
Lip thickness ratio 1	rs3790553	*WARS2*	1:119036653	NR	NR	NR	NR	NR	8.00E-09	[161]
Lip thickness ratio 2	rs3790553	*WARS2*	1:119036653	NR	NR	NR	NR	NR	9.00E-09	[161]
Segment 17	rs12890110	*RAD51B*	14:68314878	C	0.36	NR	NR	NR	3.00E-08	[169]
Segment 18	rs4675617	*SATB2-AS1, LINC01877*	2:199478026	A	0.16	NR	NR	NR	3.00E-08	[169]
	rs7373685	*EEFSEC*	3:128388177	C	0.26	NR	NR	NR	3.00E-08	[169]
	rs657913	*MIR124-1HG, MSRA-DT*	8:10025065	G	0.44	NR	NR	NR	3.00E-08	[169]
	rs12881623	*PRKCH, SLC38A6*	14:61098119	C	0.31	NR	NR	NR	3.00E-08	[169]
Segment 19	rs6568401	*RN7SKP211, LINC02836*	6:105740943	C	0.26	NR	NR	NR	4.00E-08	[169]
	rs62051935	*LINC00917, FENDRR*	16:86462256	A	0.18	NR	NR	NR	4.00E-08	[169]
Segment 30	rs74112009	*LINC02820, RASSF9*	12:85414626	NR	NR	NR	NR	NR	3.00E-08	[163]
Segment 32	rs8176501	*TFPI*	2:187488936	G	0.29	NR	NR	NR	4.00E-08	[169]
Segment 34	rs1696840	*FGFR2*	10:121584395	G	0.33	NR	NR	NR	4.00E-08	[169]
	rs13117653	*LINC01396, STX18-AS1*	4:4803727	A	0.33	NR	NR	NR	4.00E-08	[169]
Segment 35	rs2695152	*NAV3*	12:77534016	G	0.32	NR	NR	NR	4.00E-08	[169]
Segment 36	rs921119	*SIX2, LINC01121*	2:45045673	C	0.29	NR	NR	NR	4.00E-08	[169]
	rs6022641	*RNU7-14P, ZNF217*	20:53657469	C	0.34	NR	NR	NR	4.00E-08	[169]
Segment 38	rs303751	*NFIB, CDCA4P1*	9:14480822	A	0.25	NR	NR	NR	4.00E-08	[169]
	rs227727	*C17orf67, NOG*	17:56699594	T	0.44	NR	NR	NR	4.00E-08	[169]
Segment 4	rs871502	*CCDC26*	8:128851402	G	0.21	NR	NR	NR	3.00E-08	[169]
	rs1391361	*CD96*	3:111538558	T	0.32	NR	NR	NR	3.00E-08	[169]
Segment 9	rs9923447	*RPGRIP1L*	16:53609191	T	0.22	NR	NR	NR	3.00E-08	[169]
	rs7694450	*TNIP3, NDNF*	4:121076949	A	0.43	NR	NR	NR	3.00E-08	[169]

	rs2465274	*NHP2P2, HOXA1*	7:27068508	A	0.30	NR	NR	NR	3.00E-08	[169]
	rs10919462	*PRRX1, MROH9*	1:170765749	A	0.45	NR	NR	NR	4.00E-08	[169]
	rs76770688	*KCNN2, YTHDC2*	5:113649976	G	0.44	NR	NR	NR	4.00E-08	[169]
	rs1907342	*LRMDA*	10:76404010	G	0.17	NR	NR	NR	4.00E-08	[169]
	rs6578283	*KCNQ1, KCNQ1OT1*	11:2652345	G	0.30	NR	NR	NR	4.00E-08	[169]
	rs10779162	*LINC02820, RASSF9*	12:85488139	T	0.07	NR	NR	NR	4.00E-08	[169]
Upper lip height	rs2881324	*EPAS1*	2:46349755	G	0.10	NR	0.355 unit increase	NR	3.00E-08	[146]
Labial fissure width	rs56203676	*LRRTM4*	2:77553719	CAAA	0.41	NR	0.4608 unit decrease	[0.26-0.66]	2.00E-08	[154]
	rs71288019	*SACM1L*	3:45710501	C	0.45	NR	0.5144 unit increase	[0.32-0.71]	2.00E-08	[154]
Philtrum length	rs11707813	*FHIT*	3:61195644	C	0.16	NR	0.3952 unit increase	[0.23-0.56]	2.00E-08	[154]
Upper lip height	rs820217	*SAP30BP*	17:75692214	A	0.35	NR	0.2727 unit increase	[0.15-0.39]	2.00E-08	[154]
Lower lip height	rs4685943	*LINC00620*	3:5493590	T	0.01	NR	1.491 unit increase	[0.93-2.05]	2.00E-08	[154]
Right upper lip thickness	rs6656763	*NR*	Mapping not available	G	0.36	NR	0.017 unit decrease	-	3.00E-08	[146]
Philtrum width	rs255877	*MCC*	5:113417887	G	NR	NR	0.2 unit increase	[0.14-0.26]	9.00E-09	[156]
Philtrum width	rs2522825	*NHP2P2, HOXA1*	7:27072375	T	NR	NR	0.19 unit decrease	[0.13-0.26]	9.00E-09	[156]
Mouth morphology	rs970797	*MTX2, RPSAP25*	2:176247091	NR	NR	NR	NR	NR	6.00E-11	[160]
Mouth morphology principal component [illegible]	rs60159418	*PCDH7*	4:31119130	NR	NR	NR	17 unit decrease	NR	7.00E-12	[168]

Table S6. Summary of data obtained from NHGRI-EBI GWAS Catalog for SNPs that met the genome-wide significance *p*-value threshold of (5×10^{-8}) for traits in the chin/lower face region. RAF = risk allele frequency. OR = Odd ratio. CI = Confidence interval. NR: Not reported [158], [160]–[163], [169].

Reported Trait	Variant and risk allele	Mapped gene	Location	Minor Allele	RAF	OR	Beta	CI	P-value	Reference
Chin protrusion 1	rs2786116	*CRB1*	1:197364649	NR	NR	NR	NR	NR	7.00E-11	[161]
Chin protrusion 2	rs2786116	*CRB1*	1:197364649	NR	NR	NR	NR	NR	7.00E-11	[161]
Jaw protrusion 2	rs142166760	*GCC2*	2:108502395	NR	NR	NR	NR	NR	4.00E-08	[161]
	rs6950680	*CPED1*	7:121150233	NR	NR	NR	NR	NR	4.00E-08	[161]
Jaw protrusion 5	rs142166760	*GCC2*	2:108502395	NR	NR	NR	NR	NR	4.00E-08	[161]
	rs6950680	*CPED1*	7:121150233	NR	NR	NR	NR	NR	4.00E-08	[161]
Jaw slope 2	rs12472263	*LINC01965, AHCYP3*	2:104095684	NR	NR	NR	NR	NR	4.00E-08	[161]
	rs142166760	*GCC2*	2:108502395	NR	NR	NR	NR	NR	4.00E-08	[161]
Lower face flatness	rs142166760	*GCC2*	2:108502395	NR	NR	NR	NR	NR	4.00E-08	[161]
Chin dimples	rs62578082	*LMX1B, MVB12B*	9:126514762	NR	NR	1.14	NR	[1.089-1.194]	7.00E-11	[162]
	rs1194708	*LNCAROD, THAP12P3*	10:52440052	NR	NR	1.1	NR	[1.06-1.14]	7.00E-11	[162]
	rs17336368	*SUPT3H*	6:44783931	NR	NR	1.09	NR	[1.06-1.13]	7.00E-11	[162]
	rs112215522	*NR*	6:94203856	NR	NR	1.09	NR	[1.058-1.127]	9.00E-11	[162]
	rs6494598	*GREM1, SCG5*	15:32703267	NR	NR	1.22	NR	[1.188-1.256]	9.00E-11	[162]
	rs2476023	*CRB1*	1:197381909	NR	NR	1.26	NR	[1.22-1.31]	9.00E-11	[162]
	rs10504499	*EYA1*	8:71124669	NR	NR	1.29	NR	[1.240-1.346]	9.00E-11	[162]
	rs72678242	*LINC02331*	14:53788792	NR	NR	1.27	NR	[1.22-1.32]	1.00E-10	[162]
	rs17479393	*TEX41*	2:144895720	NR	NR	1.2	NR	[1.17-1.24]	1.00E-10	[162]
	rs10175706	*LINC01965*	2:104015356	NR	NR	1.17	NR	[1.14-1.21]	1.00E-10	[162]
	rs6028446	*NR*	20:39472902	NR	NR	1.15	NR	[1.12-1.18]	1.00E-10	[162]
	rs13035389	*RNU7-147P, PLCL1*	2:198801387	NR	NR	1.14	NR	[1.107-1.171]	1.00E-10	[162]
	rs59156997	*LINC01117*	2:176507119	NR	NR	1.2	NR	[1.15-1.25]	1.00E-10	[162]
	rs4237150	*GLIS3*	9:4290085	NR	NR	1.14	NR	[1.1-1.17]	2.00E-10	[162]
	rs4674676	*FARSB, MOGAT1*	2:222664393	NR	NR	1.19	NR	[1.14-1.24]	2.00E-10	[162]
	rs17275866	*LINC02268*	4:174150335	NR	NR	1.14	NR	[1.11-1.18]	2.00E-10	[162]
	rs55700449	*ZNF536*	19:30491258	NR	NR	1.18	NR	[1.13-1.22]	2.00E-10	[162]

	rs12509218	*VEGFC*	4:176666086	NR	NR	1.19	NR	[1.14-1.25]	2.00E-10	[162]
	rs11688237	*Y_RNA, ARHGAP15*	2:143802452	NR	NR	1.12	NR	[1.09-1.15]	2.00E-10	[162]
	rs7243821	*CCDC68*	18:54988301	NR	NR	1.13	NR	[1.096-1.165]	2.00E-10	[162]
	rs11650175	*CEP112*	17:65747532	NR	NR	1.12	NR	[1.09-1.16]	2.00E-10	[162]
	rs2073323	*TASP1*	20:13533787	NR	NR	1.13	NR	[1.094-1.165]	3.00E-10	[162]
	rs4868444	*MSX2, MIR4634*	5:174733110	NR	NR	1.19	NR	[1.134-1.242]	3.00E-10	[162]
	rs11768577	*SEM1*	7:96514492	NR	NR	1.35	NR	[1.303-1.402]	3.00E-10	[162]
	rs1766786	*TBX15*	1:118909877	NR	NR	1.18	NR	[1.13-1.23]	3.00E-10	[162]
	rs10962767	*RN7SL720P, BNC2*	9:16958465	NR	NR	1.11	NR	[1.08-1.14]	3.00E-10	[162]
	rs73168105	*LINC00540*	13:22139569	NR	NR	1.15	NR	[1.107-1.193]	3.00E-10	[162]
	rs6740960	*C2orf91*	2:41954539	NR	NR	1.12	NR	[1.084-1.151]	3.00E-10	[162]
	rs7167736	*ASB7*	15:100666433	NR	NR	1.14	NR	[1.1-1.18]	3.00E-10	[162]
	rs10078545	*MIR3977, RPL5P16*	5:82828371	NR	NR	1.11	NR	[1.080-1.145]	3.00E-10	[162]
	rs11713363	*RSRC1*	3:158110533	NR	NR	1.12	NR	[1.09-1.16]	3.00E-10	[162]
	rs199880074	*LINC01331*	5:74187296	NR	NR	1.12	NR	[1.08-1.16]	4.00E-10	[162]
	rs56347314	*CASC17*	17:71126965	NR	NR	1.11	NR	[1.076-1.140]	4.00E-10	[162]
	rs80067372	*TNFSF12-TNFSF13, TNFSF12*	17:7549435	NR	NR	1.12	NR	[1.080-1.150]	4.00E-10	[162]
	rs12505328	*RNU6-1096P, HAND2*	4:173456853	NR	NR	1.11	NR	[1.074-1.139]	4.00E-10	[162]
	rs11589479	*ADAM15*	1:155060832	NR	NR	1.13	NR	[1.090-1.173]	4.00E-10	[162]
	rs2133593	*LINC00578*	3:177584204	NR	NR	1.11	NR	[1.07-1.14]	4.00E-10	[162]
	rs12541121	*HAS2-AS1, MRPS36P3*	8:122089504	NR	NR	1.1	NR	[1.064-1.127]	4.00E-10	[162]
	rs7161418	*LINC02279, DICER1*	14:95013552	NR	NR	1.11	NR	[1.070-1.143]	4.00E-10	[162]
	rs1336149	*ARHGEF11*	1:156969383	NR	NR	1.09	NR	[1.062-1.125]	4.00E-10	[162]
	rs2347498	*NRG1*	8:32495664	NR	NR	1.09	NR	[1.06-1.13]	4.00E-10	[162]
	rs11001765	*LRMDA*	10:76390527	NR	NR	1.13	NR	[1.09-1.18]	4.00E-10	[162]
	rs12495832	*LINC02032, HNRNPA1P20*	3:148111690	NR	NR	1.15	NR	[1.1-1.2]	4.00E-10	[162]
	rs141753469	*MIR6074, HMGA2*	12:66003386	NR	NR	1.11	NR	[1.072-1.151]	5.00E-10	[162]
	rs145365164	*LINC02379*	4:126033497	NR	NR	1.11	NR	[1.070-1.148]	5.00E-10	[162]
	rs4958741	*CIR1P1, HAND1*	5:154488702	NR	NR	1.11	NR	[1.069-1.147]	5.00E-10	[162]
Chin morphology	rs4915551	*DENND1B*	1:197539771	NR	NR	NR	NR	NR	5.00E-10	[160]

Segment 12	rs58687115	*SLC6A15, RPL6P25*	12:83805950	C	0.49	NR	NR	NR	5.00E-08	[169]
Segment 18	rs114777090	*DPY19L2P2*	7:103261242	NR	NR	NR	NR	NR	4.00E-08	[163]
Segment 24	rs79037251	*LINC01376*	2:19303254	T	0.15	NR	NR	NR	5.00E-08	[169]
	rs10178696	*LINC01117*	2:176500891	A	0.27	NR	NR	NR	5.00E-08	[169]
	rs112087864	*THSD4*	15:71568638	T	0.31	NR	NR	NR	5.00E-08	[169]
Segment 25	rs3910659	*STX18*	4:4536189	G	0.17	NR	NR	NR	5.00E-08	[169]
Segment 26	rs9899183	*TNFSF12, TNFSF12-TNFSF13*	17:7549660	C	0.26	NR	NR	NR	5.00E-08	[169]
Segment 27	rs13035645	*MIR4432HG*	2:60406328	A	0.26	NR	NR	NR	5.00E-08	[169]
Segment 4	chr10:1582881	*NR*	Mapping not	NR	NR	NR	NR	NR	4.00E-08	[163]
Segment 48	rs17023457	*LINC01780*	1:119333107	C	0.18	NR	NR	NR	5.00E-08	[169]
	rs2245221	*TRPS1*	8:115612652	G	0.43	NR	NR	NR	5.00E-08	[169]
Segment 49	rs9893705	*BCAS3*	17:61192194	T	0.19	NR	NR	NR	4.00E-08	[169]
	rs11675008	*LINC02831, LINC01812*	2:67689743	A	0.38	NR	NR	NR	5.00E-08	[169]
Segment 51	rs577676	*PRRX1, GORAB*	1:170618199	T	0.46	NR	NR	NR	5.00E-08	[169]
	rs7513680	*PSMC1P12, RNA5SP56*	1:118608345	C	0.39	NR	NR	NR	5.00E-08	[169]
	rs2760734	*SMG6*	17:2124350	T	0.17	NR	NR	NR	5.00E-08	[169]
Segment 53	rs13035389	*RNU7-147P, PLCL1*	2:198801387	C	0.45	NR	NR	NR	5.00E-08	[169]
	rs9388518	*RPS4XP9, RSPO3*	6:126782640	A	0.44	NR	NR	NR	5.00E-08	[169]
	rs148375239	*SCG5, GREM1-AS1*	15:32710663	T	0.47	NR	NR	NR	5.00E-08	[169]
Segment 54	rs4296976	*SEM1*	7:96527536	C	0.28	NR	NR	NR	5.00E-08	[169]
Segment 6	rs56081252	*EPHB3, MAGEF1*	3:184658160	A	0.35	NR	NR	NR	5.00E-08	[169]
Chin protrusion	rs3827760	*EDAR*	2:108897145	G	NR	NR	0.0076 NR de-	NR	1.00E-79	[158]
